# Synthesis and electrokinetics of cationic spherical nanoparticles in salt-free non-polar media†
†Electronic supplementary information (ESI) available. See DOI: 10.1039/c7sc03334f. Data are also available from the Zenodo repository at DOI: 10.5281/zenodo.1066849.


**DOI:** 10.1039/c7sc03334f

**Published:** 2017-11-17

**Authors:** Gregory N. Smith, Laura L. E. Mears, Sarah E. Rogers, Steven P. Armes

**Affiliations:** a Department of Chemistry , University of Sheffield , Brook Hill , Sheffield , South Yorkshire S3 7HF , UK . Email: g.n.smith@sheffield.ac.uk ; Email: s.p.armes@sheffield.ac.uk; b Department of Chemistry , University of Liverpool , Liverpool L69 7ZD , UK; c ISIS-STFC , Rutherford Appleton Laboratory , Chilton , Oxon OX11 0QX , UK

## Abstract

Cationic diblock copolymer nanoparticles have been prepared in *n*-dodecane *via* polymerization-induced self-assembly (PISA). A previously reported poly(stearyl methacrylate)–poly(benzyl methacrylate) (PSMA–PBzMA) PISA formulation (*Chem. Sci.* 2016, **7**, 5078–5090) was modified by statistically copolymerizing an oil-soluble cationic methacrylic monomer, (2-(methacryloyloxy)ethyl)trimethylammonium tetrakis[3,5-bis(trifluoromethyl)phenyl]borate, with either SMA or BzMA, to produce either charged shell or charged core nanoparticles. The electrokinetics were studied as a function of many variables (function of volume function, particle size, solvent viscosity, and number of ions per chain). These data are consistent with electrophoresis controlled by counterion condensation, which is typically observed in salt-free media. However, there are several interesting and unexpected features of interest. In particular, charged shell nanoparticles have a lower electrophoretic mobility than the equivalent charged core nanoparticles, and the magnitude of the electrophoretic mobility increases as the fraction of cationic stabilizer chains in the shell layer is reduced. These results show that cationic PSMA–PBzMA spheres provide an interesting new example of electrophoretic nanoparticles in non-polar solvents. Moreover, they should provide an ideal model system to evaluate new electrokinetic theories.

## Abstract 2

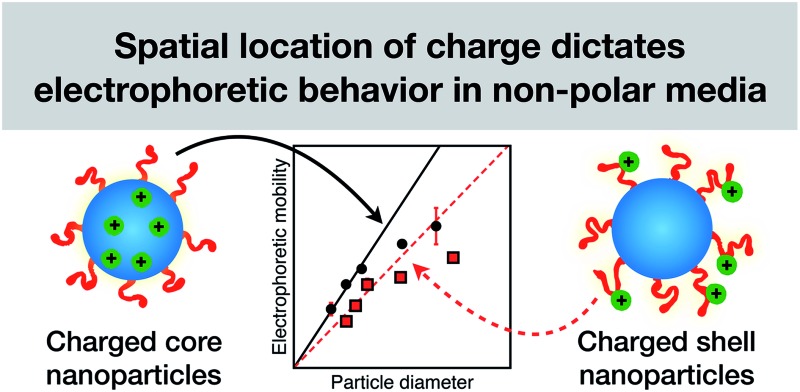
The electrokinetics of cationic sterically-stabilized diblock copolymer nanoparticles prepared in salt-free non-polar media depend on whether the charge is located in the stabilizer shell or in the nanoparticle core.

## 1 Introduction

The study of colloidal dispersions in non-polar solvents has been of long-standing academic interest.^1^–^10^ One reason for this research activity is the low relative permittivity (*ε*_r_) of the media, which results in long-range interactions. The differing length scales for ionic interactions in polar and non-polar solvents can be appreciated by considering the Bjerrum length (*λ*_B_, see eqn (1)), which is the characteristic distance at which the Coulombic attraction is equal to the thermal energy, *k*_B_*T* (*k*_B_ is the Boltzmann constant and *T* is the absolute temperature).^11^*e* is the elementary charge, and *ε*_0_ is the vacuum permittivity.1
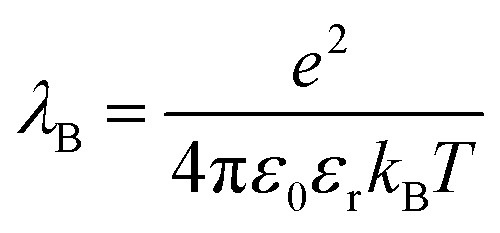



At 20 °C, *λ*_B_ for water (for which *ε*_r_ is 80.1) is 0.71 nm, whereas for non-polar solvents, such as *n*-dodecane (for which *ε*_r_ is 2.0), *λ*_B_ is 28 nm.^12^ Therefore, ions in *n*-dodecane interact over a length scale that is approximately 40 times greater than that for ions in water. Moreover, the stabilization of charged colloids in oils is pertinent to various industrial sectors, including petrochemicals,^13^,^14^ printing,^15^ and displays.^16^,^17^ Given the relatively low *ε*_r_ of non-polar solvents, producing charged particles is technically challenging and long-range interactions for such dispersions are well-known.^18^–^21^ However, when colloidally stable dispersions are produced, their electrophoretic response to an applied electric field can be exploited for various applications, such as xerography, electrophoretic displays, and electrorheological fluids.^15^–^17^,^22^–^24^ In this context, it is well-established that polymer colloids prepared in non-polar media can often acquire charge *via* the addition of an ionizable solute, such as a surfactant or small molecule salt.^20^,^25^ The addition of ionic species to particles in non-polar solvents is unlikely to be sufficient to impart colloidal stability, as would be the case for colloids in water;^19^ rather, it introduces functionality into particles that are otherwise sterically stabilized.

**Scheme 1:**
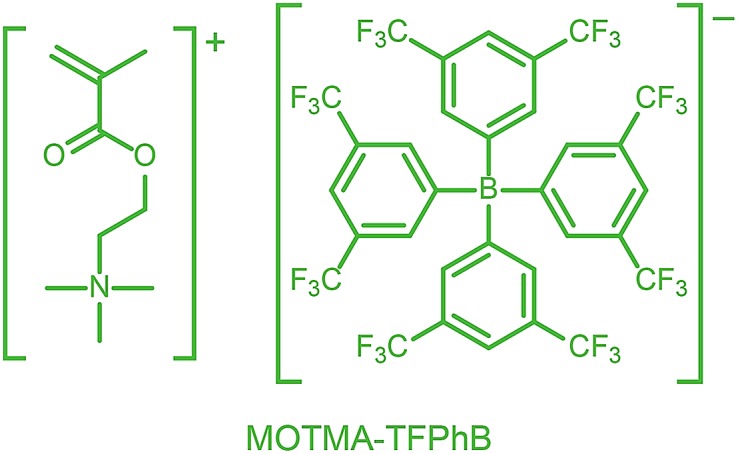
The polymerizable ionic monomer, (2-(methacryloyloxy)ethyl)trimethylammonium tetrakis[3,5-bis(trifluoromethyl)phenyl]borate (MOTMA-TFPhB) used in this work.

**Scheme 2:**
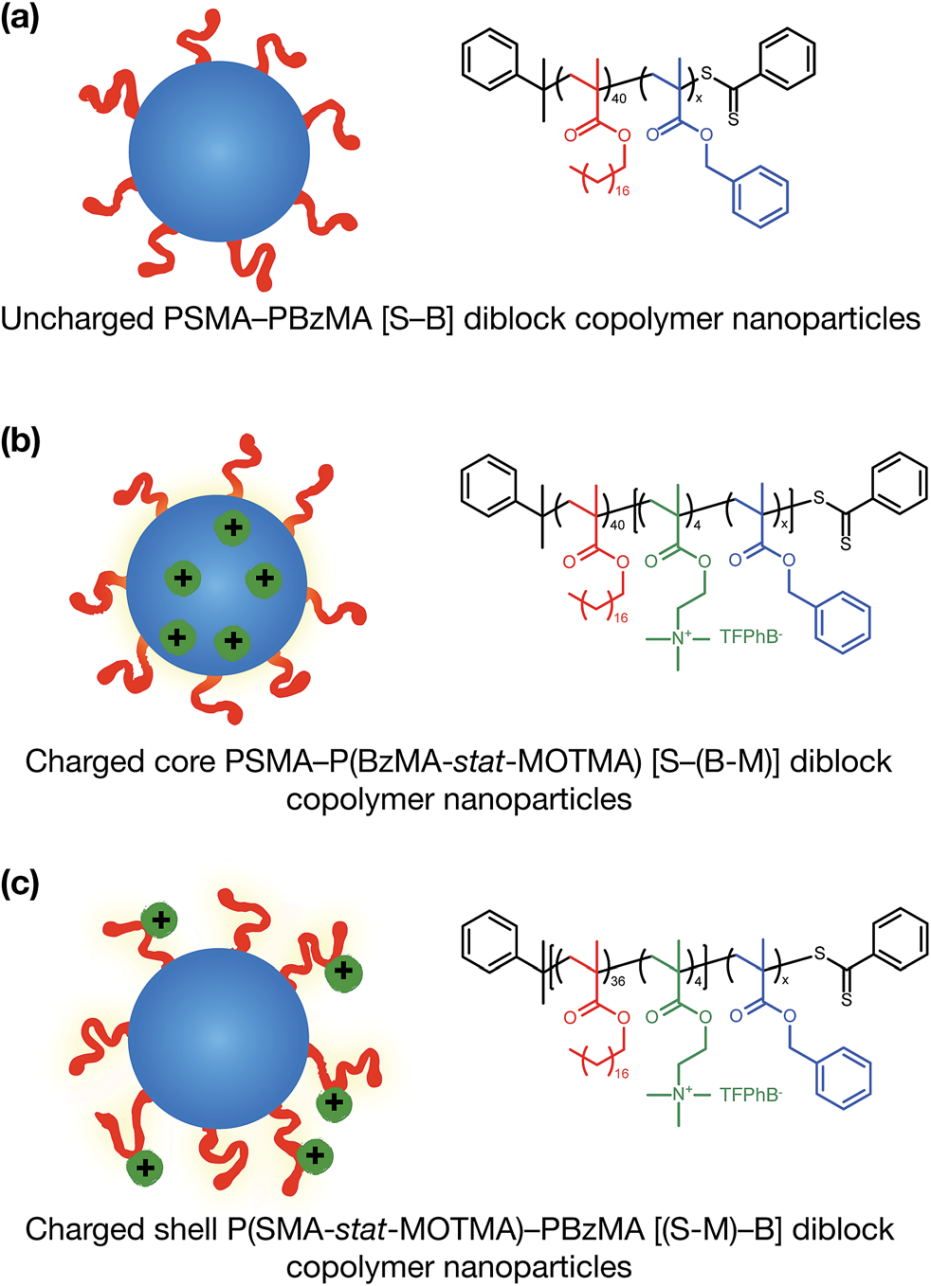
Sterically-stabilized spherical diblock copolymer nanoparticles in non-polar media prepared *via* polymerization-induced self-assembly (PISA). (a) Neutral nanoparticles (control); (b) charged core nanoparticles; (c) charged shell nanoparticles. In (b) and (c) charge is conferred by statistical copolymerization of a cationic methacrylic comonomer during the synthesis of either the core-forming or shell-forming block. Chemical structures and abbreviated names for the copolymers used in this study are also shown.

Alternatively, ionic groups can be incorporated directly into particles during the synthesis. This is the approach used in this study. The polymerizable ionic monomer, (2-(methacryloyloxy)ethyl)trimethylammonium tetrakis[3,5-bis(trifluoromethyl)phenyl]borate (MOTMA-TFPhB), shown in Scheme 1, was added during the synthesis. Particle charge arises from these surface-bound ionic groups.^26^ This approach creates a unique ionic environment, with a substantial mismatch between the highly charged particles and the ion-free non-polar solvent. This results in several characteristic features for electrokinetic measurements of charged spheres in salt-free media. Manning and Oosawa reported strong counterion condensation for polyelectrolytes in a low-salt environment,^27^,^28^ which accounts for the efficient compaction of DNA that is observed under such conditions.^29^ Ohshima has developed analytical expressions for the surface potential and electrophoretic mobility of both spheres with a charged core and also spheres and an uncharged core with a charged shell in salt-free media.^30^ A schematic representation of these different types of charged nanoparticles, in the context of the materials synthesized for this study, is shown in Scheme 2. In the high-charge limit, the electrokinetic response is moderated by counterion condensation and depends on both the volume fraction (*φ*) and the bare particle charge (*Z* = *ne*).^31^–^36^

In the literature, charged spheres have been prepared in salt-free non-polar media *via* the statistical copolymerization of an ionic monomer (with methyl methacrylate), using the Antl latex synthesis method.^2^,^37^–^39^ This is a long established method to generate polymer colloids in non-polar solvents through a straightforward synthetic protocol. Small latexes synthesized using this method are perhaps the best experimental model system for colloidal hard spheres.^40^ The poly(12-hydroxystearic acid) stabilizer copolymer, however, can vary greatly depending on the batch of precursors used.^41^ In contrast, in the present study, we have prepared sterically-stabilized methacrylic diblock copolymer spheres using reversible addition–fragmentation chain transfer (RAFT) dispersion polymerization.^42^ This formulation is an example of polymerization-induced self-assembly (PISA), which enables the preparation of diblock copolymer nano-objects directly in a solvent *via* chain extension of a soluble macromolecular RAFT agent using a suitable monomer to form an insoluble core-forming block. PISA offers several advantages compared to conventional latex polymerization: various stabilizer blocks are straightforward to synthesize, nano-objects can be prepared as concentrated dispersions without requiring purification, and nanoparticles with desired morphologies and sizes can be reproducibly targeted. The PISA approach was originally devised to produce diblock copolymer nano-objects in water^43^ but has been recently extended by us^44^–^51^ and others^52^–^58^ to non-polar solvents.^59^ PISA enables the rational design of block copolymer nano-objects with various core-forming blocks, including poly(methyl acrylate),^52^–^54^ poly(benzyl methacrylate),^44^–^47^,^49^,^51^,^58^ poly(3-phenylpropyl methacrylate),^55^–^57^ poly(benzyl acrylate),^48^ poly(*N*-2-(methacryloyloxy)ethyl pyrrolidone),^50^ and poly(phenyl acrylate)^60^ cores. There are also examples in the literature of the synthesis of polymer nano-objects using other types of reversible-deactivation radical polymerization. For example, the synthesis of poly(lauryl methacrylate)–poly(benzyl methacrylate) nano-objects by atom transfer radical polymerization has recently been reported,^61^ and ionizable cationic spherical nanoparticles have also been prepared using nitroxide-mediated radical polymerization.^62^

In this paper, we report the incorporation of the oil-soluble cationic monomer, MOTMA-TFPhB (Scheme 1), into sterically-stabilized diblock copolymer nanoparticles in non-polar solvents. The preparation of this monomer is shown in the ESI (Scheme S1).† Uncharged poly(stearyl methacrylate)–poly(benzyl methacrylate) diblock copolymer nanoparticles (see Scheme 2(a)) can be conveniently prepared in non-polar solvents *via* PISA to provide a suitable reference system.^49^,^51^ To introduce charge into these spheres, MOTMA-TFPhB is added as a comonomer to either the PSMA stabilizer block or the PBzMA core-forming block (Scheme 2(b) and (c)). Schemes showing the synthesis of these three types of diblock copolymers are shown in the ESI (Scheme S1).† The general advantages of the PISA approach are directly relevant for these ionic nanoparticles as well. In particular, nano-objects can be reproducibly prepared with a desired particle size. Additionally, the well-defined diblock copolymer architecture means that it is possible to insert ionic units into either the stabilizer chains or the nanoparticle cores of these diblock copolymer micelles. Such fine control over the spatial location of the ionic monomer has not been previously reported.

Electrokinetic studies of both charged core and charged shell PSMA–PBzMA spheres were conducted to examine the predictions of counterion condensation theories for salt-free non-polar media. Given the spatial location of the cationic monomer, the electrokinetics and solvodynamics are expected to differ for these two model systems. Moreover, as far as we are aware, there have been no previous reports of charged shell, sterically-stabilized nanoparticles in non-polar solvents. Counterion condensation in salt-free non-polar media leads to several characteristic and somewhat counterintuitive electrophoretic features compared to that observed for charged nanoparticles in the presence of electrolyte, such as surfactant charged polymer latexes in non-polar solvents.^4^,^20^,^21^ In particular, the effect of varying the volume fraction (*φ*), the spatial location of ionic groups, and the number of charges per particle lead to some unexpected electrokinetic observations that should ultimately inspire advances in the refinement of electrokinetic theories for colloidal dispersions in salt-free non-polar media.

## 2 Experimental

### 2.1 Materials

(2-(Methacryloyloxy)ethyl)trimethylammonium chloride (MOTMA-Cl, 80 wt% solution in water), stearyl methacrylate (SMA), and benzyl methacrylate (BzMA, 96%) monomers were all purchased from Sigma-Aldrich (UK). BzMA monomer was passed through a basic alumina column to remove inhibitor prior to use. Sodium tetrakis(3,5-bis(trifluoromethyl)phenyl)borate (Na-TFPhB) was a gift from Merck Chemicals Ltd (UK). 2,2-Azobisisobutyronitrile (AIBN) initiator was purchased from Molekula (UK), and *tert*-butyl peroxy-2-ethylhexanoate (T21s) initiator was a gift from AkzoNobel (The Netherlands). Cumyl dithiobenzoate (CDB, 99%) was purchased from Sigma-Aldrich (UK) and used a supplied. Solvents for synthesis and purification (dichloromethane, toluene, tetrahydrofuran, ethanol, methanol, and isopropanol) were purchased from either VWR, Sigma-Aldrich, or Fisher (UK) and were used as supplied. Deuterated solvents were obtained from either Cambridge Isotope Laboratories (USA) (acetone-*d*_6_ and dichloromethane-*d*_2_) or Sigma-Aldrich (UK) (chloroform-*d*_3_). Solvents to prepare dispersions were obtained from either Sigma-Aldrich (UK) (*n*-dodecane, ≥99%) or Alfa Aesar (UK) (*n*-hexadecane, 99%).

#### 2.1.1 Ionic monomer

The ionic comonomer (2-(methacryloyloxy)ethyl)trimethylammonium tetrakis(3,5-bis(trifluoromethyl)phenyl)borate (MOTMA-TFPhB) was obtained from a salt metathesis reaction, as previously described in the literature.^63^ Briefly, sodium tetrakis(3,5-bis(trifluoromethyl)phenyl)borate was dissolved in dichloromethane and combined with an aqueous solution of (2-(methacryloyloxy)ethyl)trimethylammonium chloride in a separating funnel. The organic layer was collected and rinsed once with water to remove the water-soluble salt byproduct. The organic phase was collected, and the solvent removed under vacuum to isolate the desired monomer. *δ*^1^H (400 MHz; acetone-*d*_6_; solvent reference) 1.95 (3H), 3.56 (9H), 4.09 (2H), 4.78 (2H), 5.74 (1H), 6.15 (1H), 7.68 (4H), 7.81 (8H). Elemental analysis. Found: C, 48.6%; H, 3.2%; N, 1.4%; Cl, 0.0%. Calc. for C_41_H_30_BF_24_NO_2_: C, 47.6%; H, 2.9%; N, 1.4%.

#### 2.1.2 Synthesis of PSMA macromolecular chain transfer agent (macro-CTA)

Non-ionic and ionic PSMA macro-CTAs were prepared similarly. For the non-ionic PSMA_40_ macro-CTA, SMA (20.0167 g, 59.1 mmol), CDB (0.4377 g, 1.61 mmol), and AIBN (0.0532 g, 0.32 mmol; CDB/AIBN molar ratio = 5.0) were dissolved in toluene (30.5538 g). The solution was purged with nitrogen and then heated at 70 °C for 10 h. The crude PSMA was purified by precipitation into ethanol to remove unreacted monomer and initiator. According to ^1^H NMR spectroscopy analysis in CDCl_3_, the polymerization reached 69% conversion. The purified polymer was also characterized using gel permeation chromatography (GPC) to determine the molar mass distribution (*M*_n_ = 11 100 g mol^–1^, *M*_w_ = 13 400 g mol^–1^, *Đ*_M_ = *M*_w_/*M*_n_ = 1.21). End-group analysis by ^1^H NMR spectroscopy in CD_2_Cl_2_ indicated a mean degree of polymerization (DP) of 40 (integrated CDB aromatic protons at 7.1–8.1 ppm were compared with the two PSMA oxymethylene protons at 3.8–4.0 ppm). For the ionic P(SMA_36_-*stat*-MOTMA_4_) macro-CTA, SMA (11.2104 g, 33.1 mmol), MOTMA-TFPhB (3.9389 g, 3.80 mmol), CDB (0.3174 g, 1.17 mmol), and AIBN (0.0403 g, 0.25 mmol; CDB/AIBN molar ratio = 5.0) were dissolved in toluene (23.7839 g). This reaction solution was purged with nitrogen and then heated at 70 °C for 10 h. Unfortunately, incorporation of the ionic monomer meant that the resulting macro-CTA could not be precipitated using excess ethanol. Instead, unreacted MOTMA-TFPhB monomer was removed by precipitation of the crude copolymer into ice-cold methanol, which is a bad solvent for both SMA and PSMA. This crude copolymer was purified by dialysis against isopropanol to remove all unreacted SMA monomer, as monitored by ^1^H NMR spectroscopy in CDCl_3_. According to ^1^H NMR spectroscopy in CDCl_3_, the polymerization of the comonomers was to 84% for PSMA and 87% for PMOTMA, suggesting similar comonomer reactivities for these two methacrylic monomers. GPC analysis was not attempted because of the likelihood of column-adsorption problems. The purified copolymer was characterized by ^1^H NMR spectroscopy in CD_2_Cl_2_, which indicated a MOTMA comonomer content of 10 mol% and a mean DP of 40 by comparing the integrated CDB aromatic protons at 7.1–8.1 ppm with the two SMA oxymethylene protons and the two MOTMA oxymethylene protons at 4.2–4.6 ppm.

#### 2.1.3 Synthesis of non-ionic and ionic PSMA–PBzMA copolymer spheres

The RAFT dispersion polymerization reactions of BzMA in *n*-dodecane was conducted at 20 wt%. BzMA, T21s initiator (added as a 10 wt% solution in *n*-dodecane; T21s/macro-CTA molar ratio = 3), and PSMA or P(SMA-*stat*-MOTMA) macro-CTA were combined using appropriate masses. Each reaction solution was purged with nitrogen and then heated at 90 °C for 18–24 h. Conversions were determined by ^1^H NMR spectroscopy in CDCl_3_, and molar mass distributions were assessed for non-ionic diblock copolymers using GPC. Characterization of all multiblock polymers is given in the ESI (Tables S1–S8).†

#### 2.1.4 Preparation of dilute dispersions of copolymer spheres

The as-synthesized spherical nanoparticle dispersions prepared at 20 wt% were diluted to volume fractions ranging from 5 × 10^–5^ to 2.2 × 10^–3^ (equivalent to 7 × 10^–3^ to 0.02 wt%) as desired using either *n*-dodecane (stored over molecular sieves) or *n*-hexadecane (used as supplied). The presence of moisture in non-polar solvents can influence their electrokinetics and electrostatics. Every effort was made to minimize exposure of these dispersions to the atmosphere. The amount of trace water in the hydrocarbon solvents used in this study was measured by Karl Fischer titration and was found to be 6 ± 1 ppm in *n*-dodecane and 20.8 ± 0.5 ppm in *n*-hexadecane.

### 2.2 Analytical methods

#### 2.2.1 Small-angle neutron scattering (SANS)

Neutron scattering measurements were performed using the instrument Sans2d at the ISIS Pulsed Neutron Source (STFC Rutherford Appleton Laboratory, Didcot, UK).^64^ The modulus of the momentum transfer vector (*Q*) is defined in eqn (2), where *θ* is half the scattering angle and *λ* is the wavelength of the radiation.2
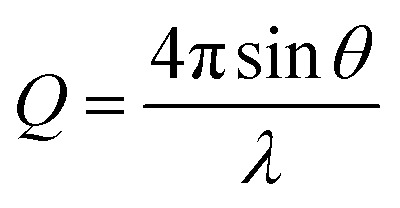



A simultaneous *Q*-range of 0.006–0.68 Å^–1^ was achieved by using an incident wavelength range of 1.75–16.5 Å and employing an instrument setup with source–sample and sample–detector distances of *L*_1_ = *L*_2_ = 4 m and the 1 m^2^ detector offset vertically 60 mm and sideways 100 mm. Raw scattering data sets were corrected for the detector efficiency, sample transmission, and background scattering and converted to scattering cross sections using the instrument-specific software, Mantid.^65^,^66^ These data were placed on an absolute scale (cm^–1^) using the scattering from a standard sample (a solid blend of hydrogenous and perdeuterated polystyrene).^67^ Data were fit to models as described in the text using the SasView small-angle scattering software package.^68^

#### 2.2.2 Dynamic light scattering (DLS)

Solvodynamic *Z*-average particle diameters were determined from cumulants analysis (Malvern Zetasizer software) using a Malvern Zetasizer Nano ZS. Diffusion coefficients (*D*) were converted to particle radii (*r*) using the Stokes–Einstein equation,^69^,^70^ where *k*_B_*T* is the thermal energy and *η* is the solvent viscosity.3
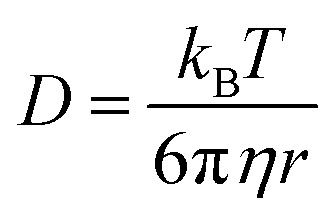



Alternatively, intensity-average size distributions were determined using the same software. These were converted to number-average (*d*_N_) and volume-average (*d*_V_) size distributions using Mie scattering theory, inputting refractive indexes of *n*-dodecane and PBzMA taken from the literature.^71^ Measurements were performed at 25 °C on dispersions with a concentration of ∼0.1 wt%. Three measurements of approximately ten runs of 10 s duration were performed and averaged.

#### 2.2.3 Transmission electron microscopy (TEM)

Diblock copolymer dispersions were diluted to generate 0.01 wt% dispersions. Copper TEM grids (Agar Scientific, UK) were surface-coated in-house to yield a thin film of amorphous carbon. Each diblock copolymer dispersion was placed onto a grid and the solvent allowed to evaporate slowly at room temperature. To stain the deposited nanoparticles, the grids were exposed to ruthenium(iv) oxide vapor for 7 min at 20 °C prior to analysis.^44^ This heavy metal compound acted as a positive stain to improve contrast. The ruthenium(iv) oxide was prepared as follows: ruthenium(ii) oxide (0.30 g) was added to water (50 g) to form a black slurry; addition of sodium periodate (2.0 g) with stirring produced a yellow solution of ruthenium(iv) oxide within 1 min. Imaging was performed at 100 kV using a Phillips CM100 instrument equipped with a Gatan 1k CCD camera. Number-average particle size distributions were obtained by measuring the area of at least 100 nanoparticles and then calculating a histogram with 5 nm wide bins using ImageJ 1.51p.^72^ These histograms were then fit to a Gaussian distribution.

#### 2.2.4 Phase-analysis light scattering (PALS)

Electrophoretic mobilities were determined from PALS measurements using a Malvern ZetaSizer Nano ZS with a universal dip cell electrode. The applied field strength was 2.0 × 10^4^ V m^–1^. Measurements were performed at 25 °C on dispersions with concentrations specified in the text. Ten runs of between 50 and 200 measurements were performed, depending on the intensity of the scattered light.

#### 2.2.5 Gel permeation chromatography (GPC)

Molecular weight distributions were assessed by GPC at 35 °C. The set-up comprised a guard column and two 5 μm PL-gel Mixed-C columns connected in series to an Agilent Technologies 1260 Infinity refractive index detector, using tetrahydrofuran containing 2.0 vol% triethylamine and 0.05 vol% butylhydroxytoluene as an eluent at a flow rate of 1.0 mL min^–1^. A series of ten near-monodisperse poly(methyl methacrylate) (PMMA) standards (*M*_p_ ranging from 1280 to 330 000 g mol^–1^) were employed as calibration standards.

#### 2.2.6 Small-angle X-ray scattering (SAXS)

Small-angle X-ray scattering (SAXS) measurements were performed on a Bruker AXS Nanostar instrument at the University of Sheffield. It was modified with microfocus X-ray tube (GeniX3D, Xenocs) and motorized scatterless slits for the beam collimation and used a 2D HiSTAR multiwire gas detector. The sample-detector distance was 1.46 m, and *λ* was of Cu Kα radiation. This gave an accessible *Q*-range (eqn (2)) of 0.008 < *Q* < 0.16 Å^–1^. Glass capillaries of 2.0 mm diameter were used as a sample holder, and an exposure time of 1.0 h was utilized for each sample. SAXS data were reduced using Nika macros for Igor Pro. SAXS data were analyzed using custom implemented spherical diblock copolymer micelle models written for the Irena package^73^ implemented in Igor Pro 6.37.

## 3 Results and discussion

PISA has been used to prepare spherical nanoparticles comprising a poly(benzyl methacrylate) (PBzMA) core block and a poly(stearyl methacrylate) (PSMA) stabilizer (or shell) block. Both charged shell and charged core polymer spheres have been prepared in a salt-free non-polar solvent, *n*-dodecane. Counterion condensation is a characteristic property of charged nanoparticles in salt-free media, particularly in low dielectric solvents.^31^–^36^ This results in effective particle charges and electrophoretic mobilities that are strongly dependent on the particle volume fraction (*φ*). The model PSMA–PBzMA spheres described in this study contain a cationic comonomer whose spatial location can be varied according to the synthesis conditions. In the following sections, we show that such charged nanoparticles exhibit various well-known features of counterion condensation along with some unexpected behavior.

**Fig. 1:**
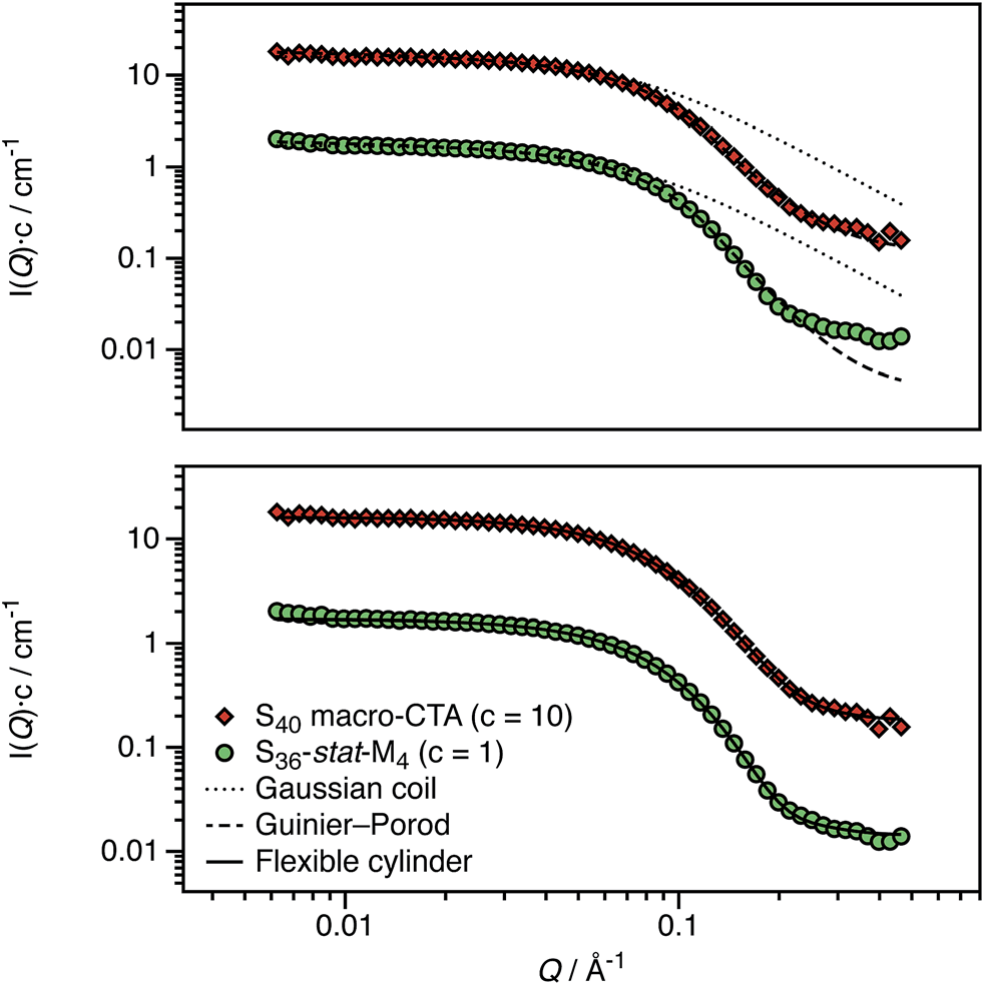
SANS scattering curves and model fits obtained for non-ionic (PSMA_40_, S_40_) and ionic (P(SMA_36_-*stat*-MOTMA_4_), S_36_-*stat*-M_4_) macro-CTAs dissolved at 2.0 wt% in *n*-dodecane-*d*_26_. The top panel shows the data compared to the scattering calculated for a Gaussian coil (dotted line), with poor agreement in the high-*Q* regime, and also for a fit to the Guinier–Porod model (dashed line), for which much better agreement is observed. The relatively long stearyl side-branches extending from the methacrylic backbone lead to a more globular structure, which results in deviation from the scattering expected for a Gaussian coil. The bottom panel shows a satisfactory data fit to a flexible cylinder model (solid line), suggesting that these macro-CTAs are best considered as “bottlebrush”-type polymers.^74^,^75^

A pair of RAFT macro-CTAs was prepared with the same overall degree of polymerization (DP) but with different functionality. A non-ionic PSMA macro-CTA was composed entirely of SMA repeat units, whereas a cationic P(SMA-*stat*-MOTMA) macro-CTA contained four MOTMA-TFPhB units in addition to SMA. Small-angle neutron scattering (SANS) measurements were performed on these two precursor blocks at a concentration of 2.0 wt% in *n*-dodecane-*d*_26_. Deuterated solvent was used to ensure sufficient isotopic contrast for SANS measurements. Both the raw data and fits to several models are shown in Fig. 1. Gratifyingly, the scattering curves obtained for these two copolymers are very similar. This confirms that 10 mol% cationic comonomer does not significantly impact their conformation in *n*-dodecane, so it is reasonable to assume that these two macro-CTAs should behave similarly when used as steric stabilizers for the PISA syntheses of diblock copolymer nanoparticles.

The radius of gyration (*R*_g_) of each polymer can be calculated using the Guinier approximation^76^ at low-*Q* (*R*_g_ ∼ 21 Å in both cases). This value was then used to calculate the scattering expected for a Gaussian coil of that *R*_g_ (dotted line in Fig. 1).^77^ The calculated scattering curves do not agree with the data, so this model is not appropriate. In view of their relatively long stearyl side-groups, the polymers were instead analyzed as “bottlebrush”-type polymers.^74^,^75^ This involved using both the Guinier–Porod model and a flexible cylinder model to fit the data, as recently reported by Pesek *et al.*^78^ The Guinier–Porod analysis^79^,^80^ (dashed line in Fig. 1) showed that both polymers had similar *R*_g_ values of 19 Å, with Porod exponents of 3.5 for the PSMA_40_ non-ionic macro-CTA and 4.0 for the P(SMA_36_-*stat*-MOTMA_4_) ionic macro-CTA. For Gaussian coils, the Porod exponent should be 2, so the above values indicate that these macro-CTAs have an interface that is more like a surface fractal (exponent between 3 and 4) or a smooth interface (exponent of 4).^81^ Both polymers are also slightly non-spherical, with a dimension parameter 3-*s* of approximately 2.92. (*s* = 0 for spheres, and *s* = 1 for rods.) Thus, these polymer chains are best considered flexible cylinders; accordingly, the SANS data have been fit to a suitable model (solid line in Fig. 1).^82^,^83^ The contour length of the cylinders was fixed (102 Å for a polymer with a mean DP of 40), and the Kuhn length and cylinder radius (assuming a Gaussian distribution) were allowed to vary. A satisfactory data fit is obtained using this model. Although the scattering curves for the two polymers are very similar, there are minor differences in the best fits. The Kuhn length is somewhat shorter for the ionic macro-CTA compared to the non-ionic macro-CTA (12 Å *versus* 18 Å), and the radius of the former is greater than the latter (16 Å *versus* 13 Å). The cylinder radii are slightly shorter compared to that expected from geometrical extension alone.^84^ In summary, SANS measurements (Fig. 1) indicate that the solution morphology of these two steric stabilizers are quite similar. Both most likely adopt a “bottlebrush”-type conformation at the surface of the corresponding respective diblock copolymer nanoparticles.

Initially, PSMA_40_–PBzMA_600_ and PSMA_40_–PBzMA_2000_ spheres were prepared in *n*-dodecane as control samples before studying the effect of introducing the cationic comonomer in different locations. The PSMA_40_ macro-CTA stabilizer has a relatively narrow molar mass distribution (*Đ*_M_ = *M*_w_/*M*_n_ = 1.22), as expected for a well-controlled RAFT solution polymerization. In contrast, both diblock copolymers have relatively broad molar mass distributions with *Đ*_M_ = *M*_w_/*M*_n_ = 1.89 and *Đ*_M_ = *M*_w_/*M*_n_ = 2.94, respectively. Gradual loss of RAFT control over the polymerization when targeting longer core-forming blocks has been previously reported for other closely-related PISA formulations.^49^ However, it is emphasized that this does not have any discernible impact on the copolymer morphology: uniform spherical nanoparticles can still be obtained.

**Fig. 2:**
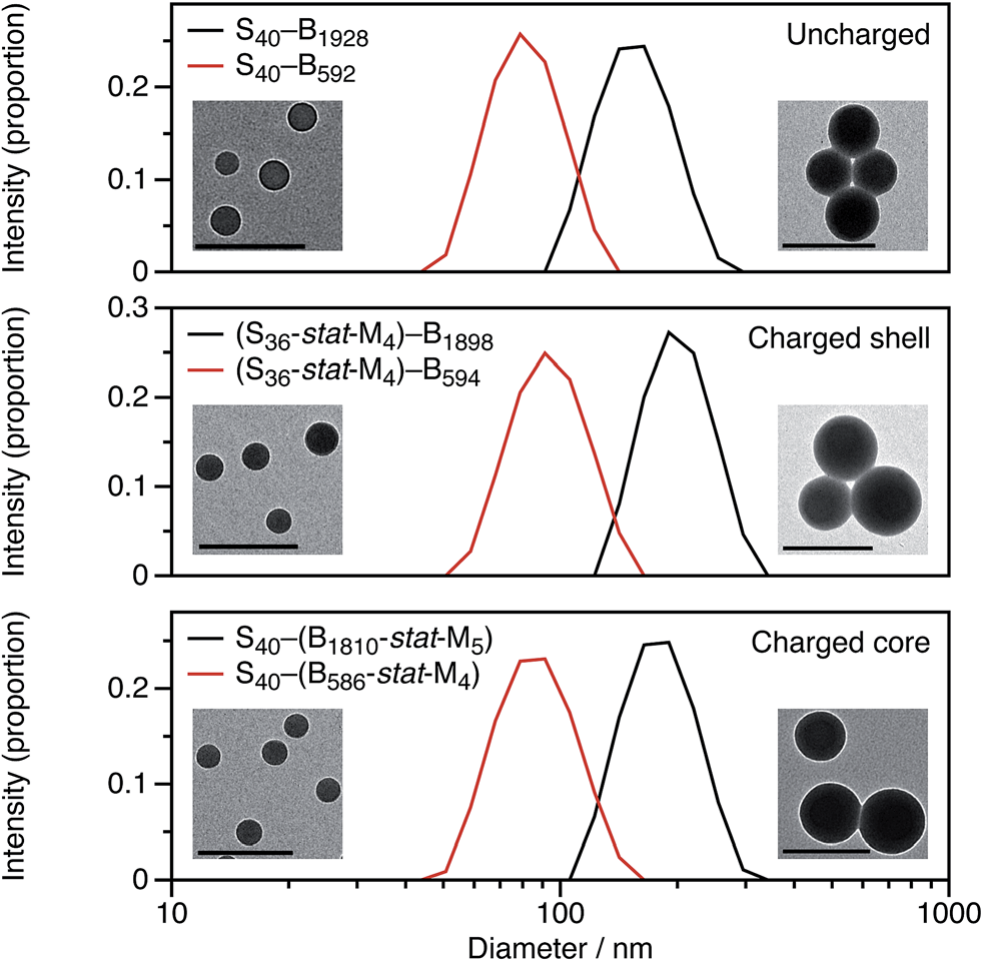
Intensity-average particle size distributions determined by DLS for uncharged, charged core, and charged shell PSMA–PBzMA diblock copolymer micelles. As expected, spheres with BzMA_2000_ cores are larger than those with BzMA_600_ cores. Adding cationic comonomer to the copolymer chains increases the particle size compared to the corresponding non-ionic copolymer chains. Moreover, the spatial location of this cationic comonomer also influences the particle size of the spheres: charged shell are larger than charged core nanoparticles. Representative TEM micrographs for the diblock copolymer nanoparticles are shown as insets. The scale bars represent 200 nm.

For these SMA-based macro-CTAs, incorporating the cationic comonomer into the stabilizer chains had little impact on their solution morphology. In contrast, the presence and location of the cationic comonomer has a strong influence on the size of the spherical nanoparticles. Intensity-average particle size distributions determined by DLS and TEM micrographs are shown in Fig. 2. Although the molar mass distributions of the diblock copolymer chains are almost certainly broad, it is emphasized that the particle size distributions are relatively narrow. The DLS polydispersity indexes for these nanoparticles are all 0.05 or below.

Fig. 2 shows DLS particle size distributions as well as TEM micrographs for uncharged, charged shell, and charged core nanoparticles targeting either PBzMA_600_ or PBzMA_2000_ cores. Incorporating the cationic comonomer into either the stabilizer block or the core-forming block always leads to larger nanoparticles. The number of cationic comonomer residues per copolymer chain is the same for the charged core and charged shell nanoparticles, and incorporating charge into the stabilizer shell has a greater effect on the particle size than copolymerizing cationic comonomer into the core-forming block. The particle size effects will be discussed in more detail in Section 3.2.

### 3.1 Varying the nanoparticle volume fraction

One consequence of counterion condensation is that electrokinetic phenomena no longer depend on the bare particle charge (*Z*) in the high-charge limit.^85^ Instead, the electrophoretic mobility (*μ*) is a function of the particle volume fraction *φ*.^39^ All electrophoresis data are shown in terms of the reduced (unitless) electrophoretic mobility, *μ*/*μ*_0_, where *μ*_0_ = *e*/(6π*ηλ*_B_)^39^ Ohshima derived an analytical expression to relate *μ* to *φ* in the high-charge limit.^33^

4 *μ*/*μ*_0_ = ln(1/*φ*)*Ω*

5
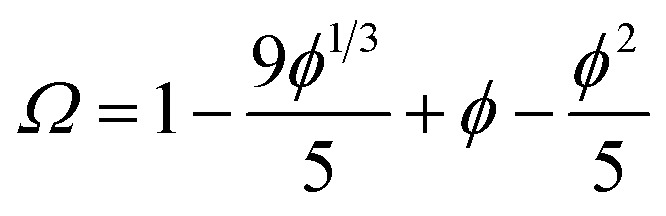



This expression is independent of particle size and represents the maximum electrophoretic mobility that can be obtained for a charged particle undergoing counterion condensation at a given volume fraction. This equation is strictly only applicable to charged core spheres.^33^ For charged shell spheres, a similar expression can be obtained that includes a contribution from the drag coefficient (*D*_H_) of a particle with a solvent-permeable shell.^35^6
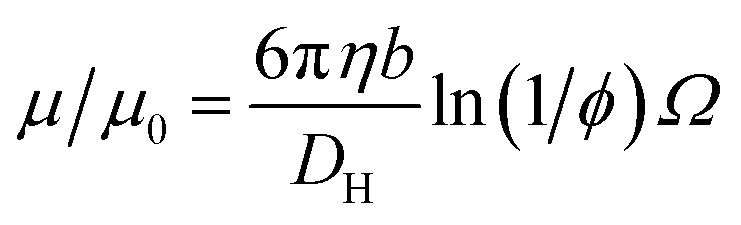



*D*_H_ can be calculated as a function of the core radius (*a*), the core radius plus shell thickness (*b*), the friction coefficient (*γ*), and the solvent viscosity (*η*). In the upper limit where *γ* → *∞*, *D*_H_ is equal to 6π*ηb*, and eqn (6) reduces to eqn (4). In the lower limit where *γ* → 0, *D*_H_ is equal to 6π*ηa*, and the reduced electrophoretic mobility of a charged shell sphere should be that of a charged core sphere multiplied by *b*/*a*.^35^ The possible effect of solvodynamics will be explored in more detail in Section 3.3 by dispersing both charged core and charged shell nanoparticles in similar solvents of differing viscosities (*n*-dodecane *versus n*-hexadecane).

**Fig. 3:**
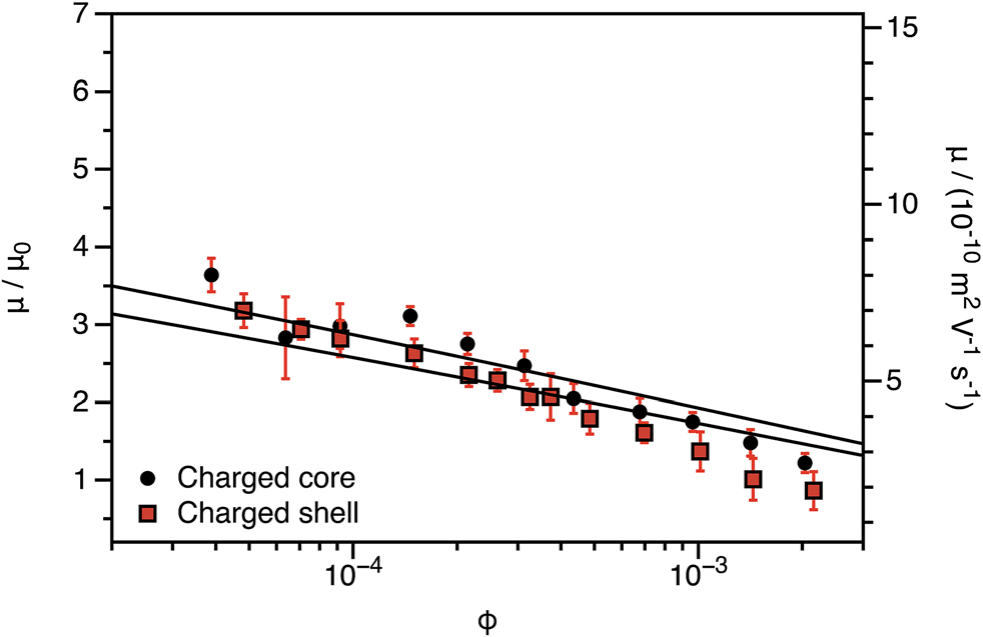
Electrophoretic mobility (*μ*) and reduced mobility (*μ*/*μ*_0_) determined for charged core and charged shell PBzMA nanoparticles (DP = 600) containing four units of cationic comonomer in either the shell or the core. The data are fitted to eqn (7) with *ν* as the only fitting parameter (solid lines). Owing to the relatively small size of the nanoparticles, *μ*/*μ*_0_ is significantly lower than that predicted (eqn (4)). Moreover, *μ*/*μ*_0_ is slightly lower for the charged shell nanoparticles compared to the charged core nanoparticles.

**Fig. 4:**
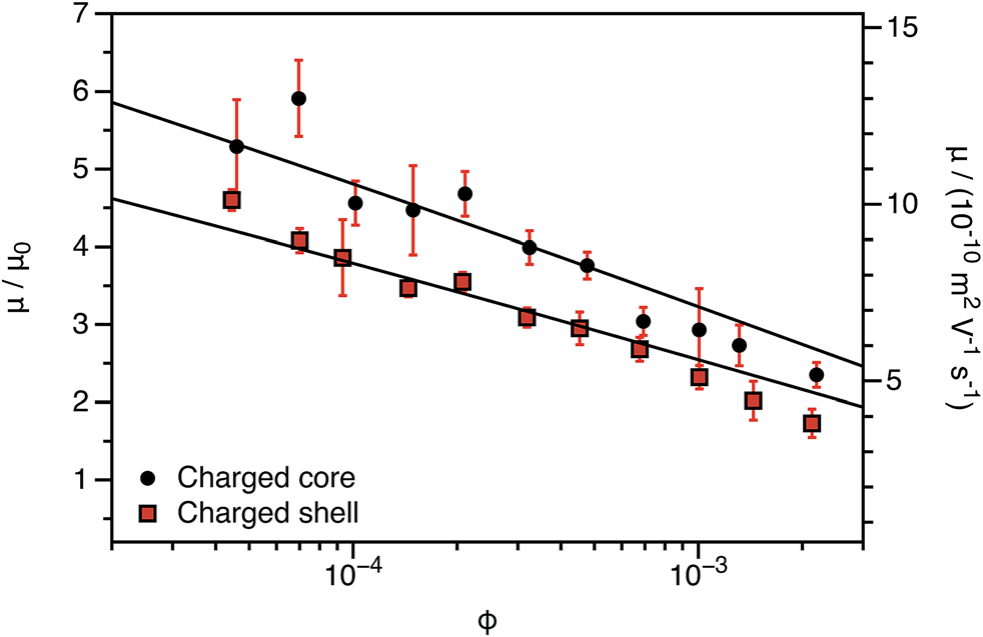
Electrophoretic mobility (*μ*) and reduced mobility (*μ*/*μ*_0_) determined for charged core and charged shell PBzMA nanoparticles (DP = 2000) containing four units of cationic comonomer in either the shell or the core. The data are fitted to eqn (7) with *ν* as the only fitting parameter (solid lines). As the nanoparticle DLS diameters (charged core: 171 nm, charged shell: 190 nm) are significantly greater than that of the nanoparticles shown in Fig. 3 (charged core: 81 nm, charged shell: 89 nm), *μ*/*μ*_0_ is closer to the maximum (eqn (4)). There is also clearly a qualitative difference between the charged core and charged shell nanoparticles, with the former exhibiting higher electrophoretic mobilities at all volume fractions (*φ*).

The experimentally-determined electrophoretic mobilities for both charged core and charged shell nanoparticles with core-forming blocks of DP 600 or 2000 are shown in Fig. 3 and 4, respectively. The magnitude of *μ*/*μ*_0_ differs for these two systems, which is not accounted for by either expression discussed above. However, similar observations have been previously reported for charged PMMA latexes in salt-free media.^39^ This indicates that this is a generic feature of the electrokinetics of charged nanoparticles in salt-free media, rather than merely an esoteric system-dependent observation.

To account for this reduction in the magnitude of *μ*/*μ*_0_, we introduce a size-dependent constant (*ν*) into eqn (4).

7 *μ*/*μ*_0_ = *ν* ln(1/*φ*)*Ω*

This is an empirical scaling parameter that modifies the magnitude of the *μ*/*μ*_0_ without affecting the *φ* dependence. The data shown in Fig. 3 and 4 have been fitted using eqn (7). For charged core nanoparticles, the numerical value of *ν* is a direct consequence of the particle diameter, similar to those reported by Gillespie *et al.* for charged PMMA latexes.^39^ For charged shell spheres, the value of *ν* is a compound function of both the particle size and drag coefficient. Thus, this provides a convenient means of quantifying how the charged shell nanoparticles differ from the charged core nanoparticles.

The agreement between the experimental data and fit values of *μ*/*μ*_0_ for the charged core spheres in Fig. 3 and 4 is very gratifying given that the only fitting parameter is the *φ*-independent magnitude *ν*. This clearly shows that the effective charge of the particles is dictated by counterion condensation. It also shows that these two dispersions are still in the high-charge limit, as the electrophoretic mobility should become independent of *φ* in the low-charge limit.^32^,^33^ However, the reason for the difference in the magnitude of *μ* for charged core and charged shell differs is not immediately apparent. Possible explanations will be considered in Section 3.4.

### 3.2 Varying the nanoparticle diameter

**Fig. 5:**
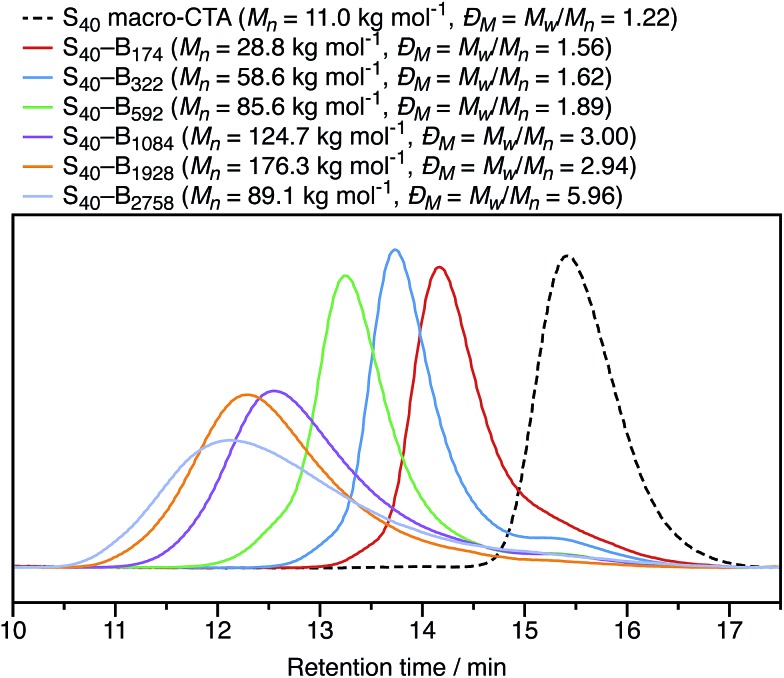
THF GPC chromatograms recorded for non-ionic PSMA_40_–PBzMA_*x*_ diblock copolymers with value of *x* ranging from 174 to 2758. The PSMA_40_ macro-CTA has a relatively low molar mass distribution (*Đ*_M_ = *M*_w_/*M*_n_ = 1.22), as do the three diblock copolymers with core-forming block DPs below 600 (*Đ*_M_ = *M*_w_/*M*_n_ < 2.00). However, higher DPs lead to progressively broader molar mass distributions (*Đ* = *MM*_w_/*M*_n_ 3.00).

**Fig. 6:**
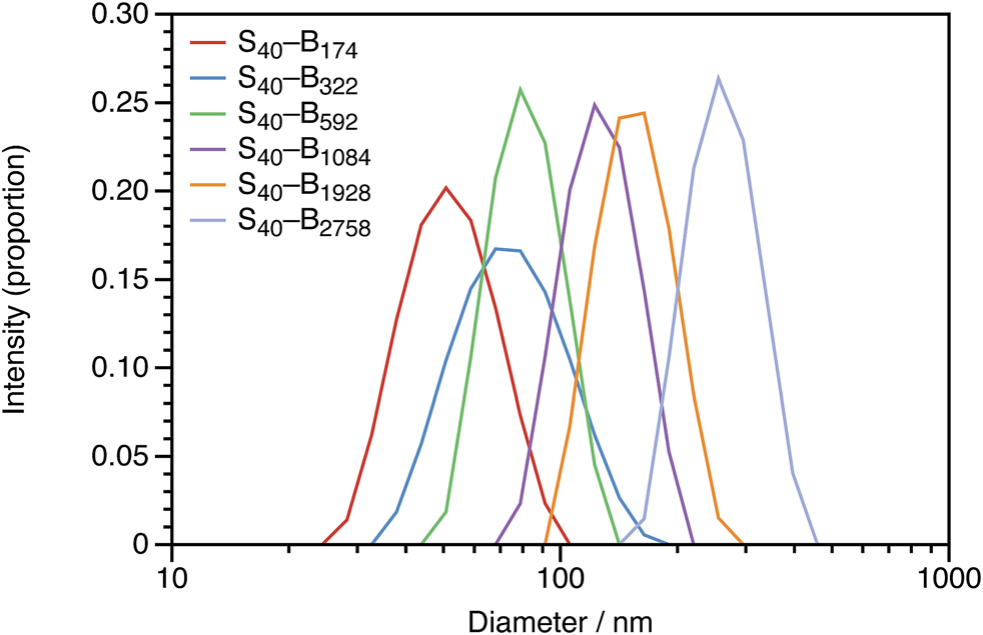
Intensity-average DLS particle size distributions obtained for uncharged PSMA_40_–PBzMA_*x*_ diblock copolymer nanoparticles with *x* values ranging from 174 to 2758. The nanoparticle diameter (*d*_Z_) increases monotonically with the DP of the core-forming block, and the polydispersity indexes are low.

In Section 3.1, the mean particle diameter was varied by adjusting the DP for the core-forming PBzMA core block. As shown in Fig. 2, increasing this target DP leads to larger nanoparticles. To further explore the effect of varying this parameter, a series of six nanoparticles were synthesized with target PBzMA DPs ranging from 180 to 3000. Relatively high conversions were obtained in all cases. GPC data for the non-ionic diblocks are shown in Fig. 5. *Đ*_M_ = *M*_w_/*M*_n_ values increase from 1.56 for PSMA_40_–PBzMA_174_ up to 5.96 for PSMA_40_–PBzMA_2758_. Nevertheless, the corresponding DLS particle size distributions remain narrow for these non-ionic diblock copolymers (Fig. 6).

Mean particle diameters were calculated for uncharged, charged shell, and charged core PSMA–PBzMA nanoparticles for core-forming block DPs ranging from ∼200 to ∼3000. Particle diameters were measured using DLS (*d*_Z_ and *d*_N_) and TEM (*d*_N_), and these data are summarized in Table 1. Derry *et al.* have previously shown that the DLS diameter has a power law dependence on DP for a series of uncharged PSMA–PBzMA spherical nanoparticles.^49^ The power law exponent *α* varies between from 0.50 to 1.0, with the lower value indicating unperturbed chains and the higher value indicating fully-extended chains. The same analysis was performed for the DLS and TEM data obtained in the present study for the uncharged, charged shell, and charged core nanoparticles. The largest spheres (DP ∼ 2000) were excluded from the fitted data in Table 1 given their relatively high *Đ*_M_ values. The *α* values for otherwise identical dispersions vary for the differently weighted diameters, so it is important to compare using a single sizing method. For values of *d*_Z_ measured by DLS for uncharged nanoparticles, *α* is equal to 0.48; this is expected for relatively long PSMA stabilizer chains, according to the literature.^49^ The value of *α* for the charged nanoparticles is typically greater than that for the uncharged nanoparticles, which suggests that the core-forming chains are weakly perturbed. This illustrates the impact of incorporating ionic groups into a low dielectric environment; just four cationic comonomer residues per copolymer chain leads to sufficient mutual repulsion.

**Table 1:** Particle sizes of diblock copolymer nanoparticles from DLS, TEM, and SAXS^*a*^

DLS and TEM	*d*_Z_ (DLS)/nm (Polydispersity index)	*d*_N_ (TEM)/nm (*c*_V_)	*d*_N_ (DLS)/nm (*c*_V_)
**Uncharged**			
S_40_–B_174_	50 (0.05)	25 (0.24)	39 (0.23)
S_40_–B_322_	71 (0.10)	36 (0.20)	50 (0.26)
S_40_–B_592_	80 (0.02)	54 (0.25)	66 (0.22)
S_40_–B_1084_	124 (0.01)	77 (0.23)	106 (0.23)
S_40_–B_1928_	159 (0.01)	109 (0.13)	143 (0.24)
S_40_–B_2758_	259 (0.02)	216 (0.15)	242 (0.24)
*α*	0.48	0.61	0.56

**Charged shell**			
(S_36_-M_4_)–B_164_	66 (0.23)	28 (0.24)	37 (0.28)
(S_36_-M_4_)–B_311_	77 (0.13)	39 (0.23)	51 (0.28)
(S_36_-M_4_)–B_594_	93 (0.01)	60 (0.27)	77 (0.22)
(S_36_-M_4_)–B_1005_	133 (0.03)	98 (0.27)	117 (0.23)
(S_36_-M_4_)–B_1898_	193 (0.03)	150 (0.25)	178 (0.25)
(S_36_-M_4_)–B_3072_	285 (0.04)	243 (0.23)	268 (0.24)
*α*	0.44	0.71	0.65

**Charged core**			
S_40_–(B_169_-M_4_)	46 (0.01)	30 (0.23)	38 (0.21)
S_40_–(B_338_-M_4_)	65 (0.05)	39 (0.24)	51 (0.24)
S_40_–(B_586_-M_4_)	84 (0.05)	53 (0.24)	68 (0.23)
S_40_–(B_1076_-M_4_)	122 (0.03)	79 (0.29)	104 (0.24)
S_40_–(B_1810_-M_4_)	178 (0.01)	142 (0.21)	163 (0.25)
S_40_–(B_3568_-M_5_)	382 (0.03)	328 (0.15)	366 (0.25)
*α*	0.56	0.65	0.61

SAXS	*d*_V_ (SAXS)/nm (*c*_V_)	*n*_agg_	*d*_V_ (DLS)/nm (*c*_V_)
S_40_–B_174_ (uncharged)	27 (0.12)	243	45 (0.27)
(S_36_-M_4_)–B_164_ (charged shell)	30 (0.13)	331	48 (0.42)
S_40_–(B_169_-M_4_) (charged core)	30 (0.11)	301	43 (0.24)

^*a*^All DLS diameters are the solvodynamic size. TEM and SAXS diameters are the core size. The coefficient of variation (*c*_V_) is given by the standard deviation over the mean (*σ*/*d*). Values of *α* (*d ∝* DP^*α*^) exclude the highest DP copolymer micelle.

In addition to DLS and TEM, small-angle X-ray scattering (SAXS) is a powerful method for characterizing nanostructures.^86^ SAXS measurements were obtained for the smallest diblock copolymer nanoparticles, and the data were fit to a spherical micelle model,^87^,^88^ as discussed in the ESI.† The advantage of using SAXS to assess nanoparticle size is that the technique is far more statistically robust than TEM and provides significantly more structural information than DLS (such as mean particle core diameters and aggregation numbers). Fits to the data are shown in the ESI,† and the best fit dimensions are shown in Table 1. The mean core diameters are similar, as would be expected for diblock copolymer nanoparticles prepared with the same core DP, although the two charged nanoparticles are larger than the uncharged nanoparticles. The core diameter is approximately 10 nm smaller than the DLS-determined *d*_V_, which suggests that the PSMA stabilizers may extend farther into the solvent than might be expected based on their radius of gyration alone. SAXS analysis also enables the determination of the aggregation number (*n*_agg_) from the spherical volume of the core divided by the volume of a single core-forming block. This is important, because it allows the number of bare charges per nanoparticle to be calculated. As expected, the larger core volume of the charged nanoparticles corresponds to higher aggregation numbers compared to that for the uncharged nanoparticles.

**Fig. 7:**
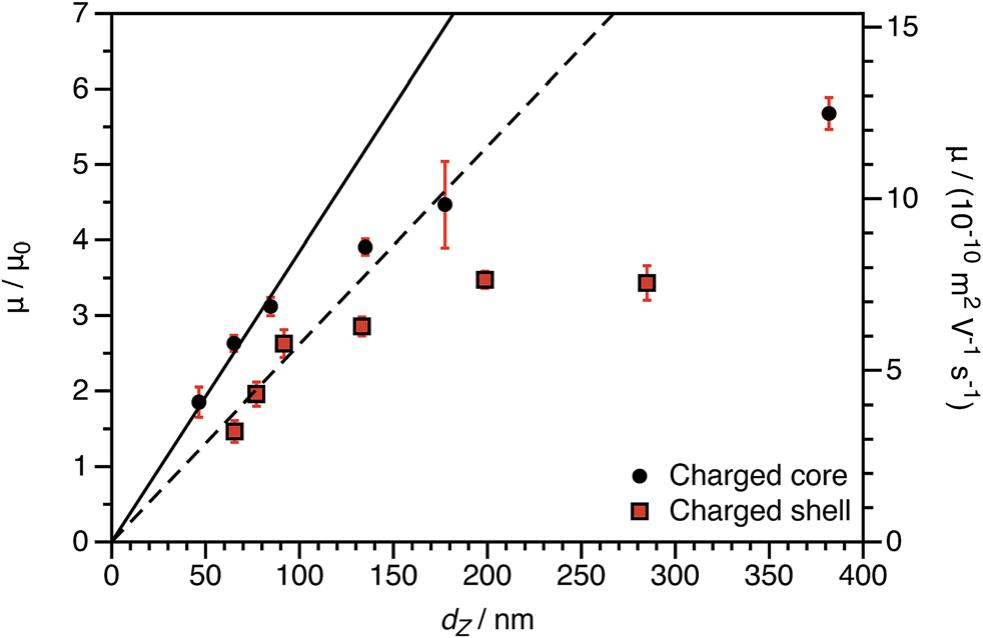
Electrophoretic mobility (*μ*) and reduced mobility (*μ*/*μ*_0_) for both charged core and charged shell nanoparticles as a function of DLS particle diameter (*d*_Z_) at a fixed volume fraction *φ* of 1.5 × 10^–4^. The linear fits to the data are for particles smaller than 100 nm (solid line: charged core, dashed line: charged shell). The magnitude of the reduced mobility increases linearly for small nanoparticles and approaches a plateau value for larger nanoparticles. From these data, it is also clear that the charged shell nanoparticles possess lower reduced mobilities than charged core nanoparticles of equivalent size.

By producing a series of nanoparticles of varying dimensions, the effect of particle size on electrophoretic mobility can be investigated. Studies of other charged core particles in non-polar solvents indicate that *μ* increases linearly with size for small particles but reaches a plateau value for larger particles.^39^ Reduced mobilities determined for both charged core and charged shell nanoparticles are shown in Fig. 7; these data are broadly consistent with the literature. The influence of the spatial location of the ionic comonomer is more discernible from this data set than from the electrophoretic data shown in Fig. 3 and 4. For particles of equivalent size, the charged shell nanoparticles possess lower mobilities than the charged core nanoparticles. This also indicates an important advantage of using PISA to produce charged nanoparticles in non-polar solvents. Compared to conventional latex syntheses based on free radical polymerization, it is rather straightforward to produce relatively small nanoparticles using RAFT-mediated PISA. Moreover, in this regime electrophoretic mobility depends most strongly on particle size. The linear fits to the data are for particles smaller than 100 nm (solid line: charged core, dashed line: charged shell). The magnitude of the reduced mobility increases linearly for small nanoparticles and approaches a limiting value for larger nanoparticles. From these data, it is also clear that the charged shell nanoparticles possess lower reduced mobilities than charged core nanoparticles of equivalent size.

### 3.3 Varying the solvent viscosity

In principle, varying the solvent viscosity should indicate whether solvodynamics are important for the electrophoresis of these sterically-stabilized nanoparticles in salt-free media. For charged core nanoparticles, the reduced mobility should be equivalent in different solvents as the *μ*_0_ term accounts for the difference in solution viscosity. However, the effect of changing the viscosity cannot be predicted *a priori* for charged shell nanoparticles. This is because the drag term (*D*_H_) in eqn (6) depends on the solvent viscosity.^35^

*n*-Hexadecane was chosen for comparison to *n*-dodecane because it is chemically very similar (essentially identical relative permittivity^12^) but has a significantly greater viscosity (approximately twice as viscous^89^).^90^ As-synthesized charged core (PSMA_40_ macro-CTA) or charged shell (P(SMA_36_-*stat*-MOTMA_4_ macro-CTA) dispersions prepared in *n*-dodecane were diluted to a volume fraction of 1.5 × 10^–4^ using either *n*-dodecane or *n*-hexadecane. Although there will be some *n*-dodecane present, at this level of dilution, the residual *n*-dodecane in dispersions in *n*-hexadecane is only around 0.1 wt%, which is considered negligible. The volume fraction was fixed to ensure that there were no differences due to counterion condensation (see eqn (4) and (6)). DLS diameters were also determined in *n*-hexadecane, and in general very similar sizes were obtained in these two solvents.

**Fig. 8:**
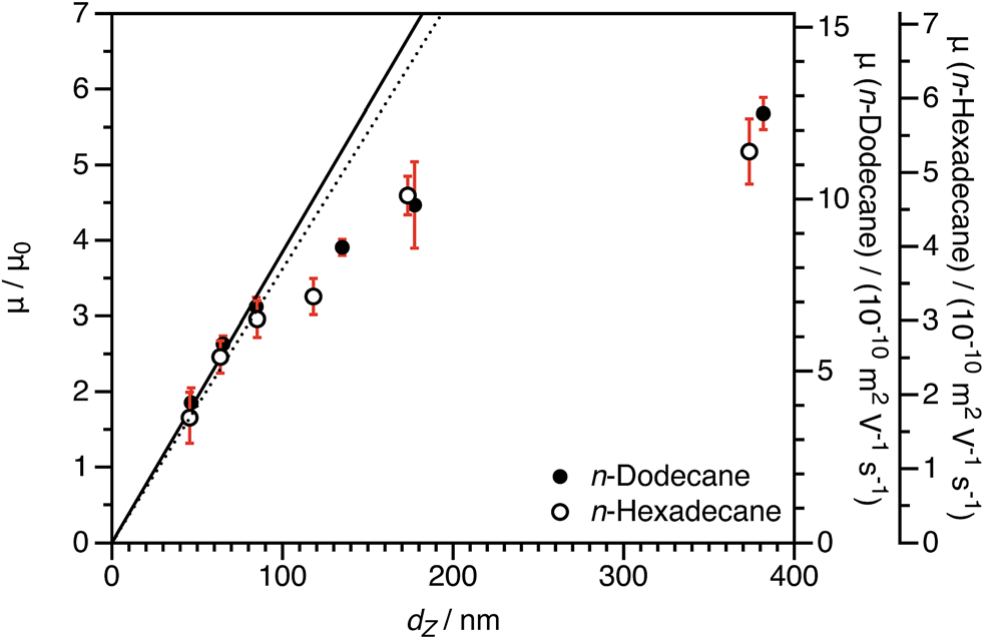
Electrophoretic mobility (*μ*) in *n*-dodecane and *n*-hexadecane and reduced mobility (*μ*/*μ*_0_) for charged core PBzMA_2000_ spheres in *n*-dodecane and *n*-hexadecane as a function of DLS particle diameter (*d*_Z_) at a fixed volume fraction *φ* of 1.5 × 10^–4^. The linear fits to the data are for particles smaller than 100 nm (solid line: *n*-dodecane, dotted line: *n*-hexadecane). The size dependence of the mobility is essentially the same in the two solvents, demonstrating that normalization by *μ*_0_ accounts for the effect of solvent viscosity.

The normalized electrophoretic mobilities for charged core nanoparticle dispersions are comparable for *n*-dodecane and *n*-hexadecane (Fig. 8). The raw experimental values of *μ* are approximately halved in *n*-hexadecane compared to *n*-dodecane. This is expected given the relative solvent viscosities.^90^ This is important, because it confirms that normalization by *μ*_0_ accounts for the influence of the solvent viscosity.

**Fig. 9:**
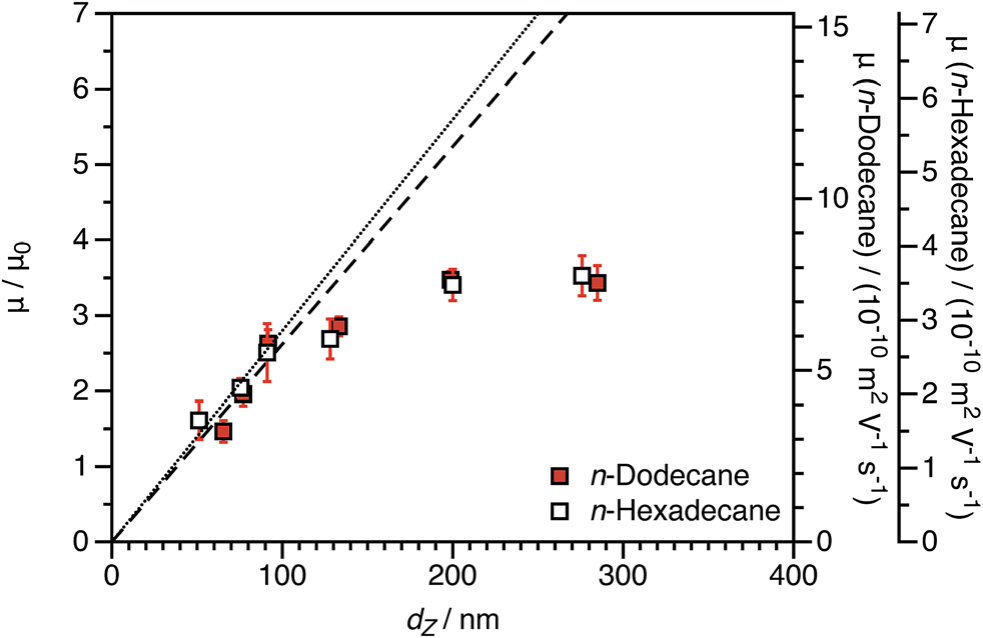
Electrophoretic mobility (*μ*) in *n*-dodecane and *n*-hexadecane and reduced mobility (*μ*/*μ*_0_) for charged shell PBzMA_2000_ spheres in *n*-dodecane and *n*-hexadecane as a function of DLS particle diameter (*d*_Z_) at a fixed volume fraction *φ* of 1.5 × 10^–4^. The linear fits to the data are for particles smaller than 100 nm (dashed line: *n*-dodecane, dotted line: *n*-hexadecane). The size dependence of the mobility is the same for the two solvents, which demonstrates that the reduction in mobility is not due to hydrodynamics in the stabilizer layer but rather due to electrostatics.

Electrophoretic mobilities for charged shell nanoparticles in *n*-dodecane and in *n*-hexadecane are shown in Fig. 9. As for charged core nanoparticles shown in Fig. 8, the viscosity-normalized mobilities are essentially identical in these two solvents. The magnitude of *μ*/*μ*_0_ is less than for the charged core spheres in both solvents. This strongly suggests that the reduction in mobility for charged shell nanoparticles, discussed in Section 3.1, is not due to solvodynamics (which would be influenced by the change in viscosity) but rather by electrostatics.

### 3.4 Varying ionic fraction

For both charged core and charged shell nanoparticles in salt-free media, the onset of counterion condensation occurs at a certain particle charge (*Z*).^32^,^33^,^85^ Above this critical value, the electrophoretic mobility is independent of the particle charge and is proportional to *φ* (eqn (4)). Below this critical value, the electrophoretic mobility depends on the particle charge. As far as we are aware, all reports of charged spheres in salt-free non-polar solvents lie within the high-charge limit,^37^–^39^ so it is not possible to determine the effect of bare particle charge. By using PISA to synthesize these charged nanoparticles, the number of charged groups can be controlled with high precision for both charged core and charged shell nanoparticles. To tune the nanoparticle charge density, the proportion of cationic comonomer copolymerized with PBzMA during PISA was lowered. To reduce the charge density for the charged shell spheres, a binary mixture comprising non-ionic PSMA_40_ and cationic P(SMA_36_-*stat*-MOTMA_4_) was used to confer steric stabilization, as described elsewhere.^91^–^93^

**Fig. 10:**
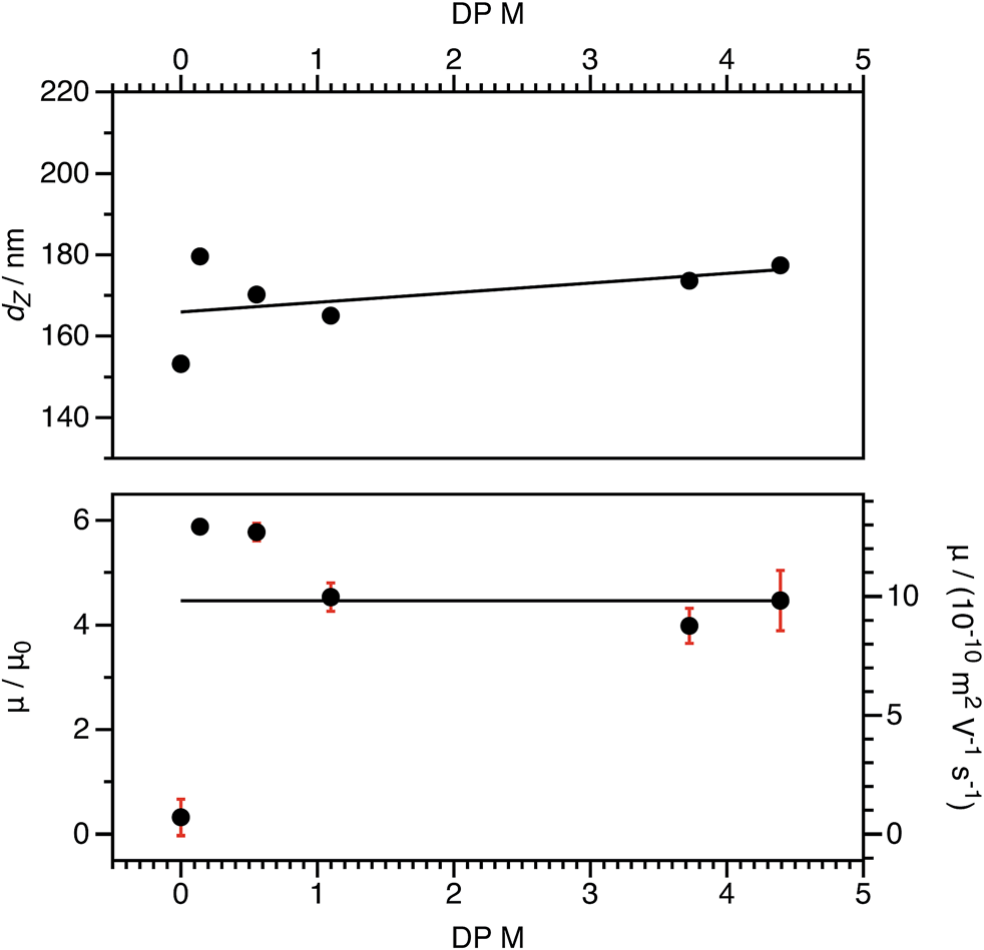
DLS solvodynamic diameters (*d*_Z_, top), electrophoretic mobility (*μ*, bottom), and reduced mobilities (*μ*/*μ*_0_, bottom) for charged core PSMA_40_–(PBzMA_2000_-*stat*-PMOTMA_*m*_) nanoparticles in *n*-dodecane at a fixed volume fraction (1.5 × 10^–4^). The diameters vary slightly as the content of the cationic MOTMA comonomer is systematically reduced; fewer ionic groups result in smaller nanoparticles. (The solid line is a linear fit as a guide to the eye.) However, the electrophoretic mobility, remains independent of the cationic comonomer content. (The solid line represents the mean value.) This suggests that an extremely small number of ionic groups would be required to access the low-charge limit in non-polar solvents.

For charged core spheres, the cationic comonomer content was reduced from 0.20 mol% (relative to BzMA) to 0.01 mol%. This is equivalent to one cationic charge per seven copolymer chains (or per 14 000 BzMA units). From the electrophoresis data shown in Fig. 10, the resulting nanoparticles are nevertheless still appreciably cationic. The cationic charge appears to increase for very low cationic comonomer contents, but it is not clear whether this is a real effect or simply reflects scatter in the data. As expected, there is a concurrent size reduction for lower proportions of cationic comonomer. The size and electrophoretic mobility of the equivalent uncharged nanoparticles are also shown in Fig. 10 as a reference. These nanoparticles have an effective zero charge, and their mean radius is less than that of the charged core nanoparticles. These data suggest that it will be technically challenging to access the low-charge limit in non-polar solvents, because a remarkably small number of cationic comonomer units still result in highly charged nanoparticles.

**Fig. 11:**
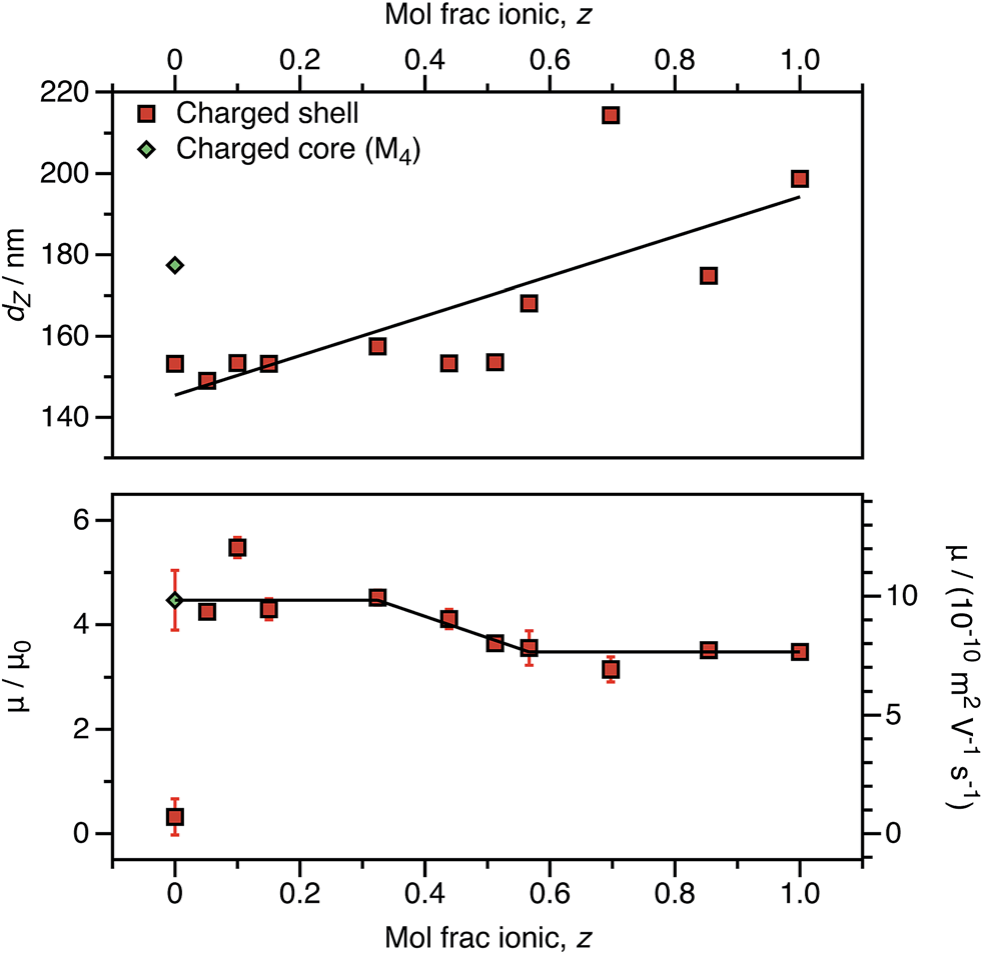
DLS solvodynamic diameter (*d*_Z_, top), electrophoretic mobility (*μ*, bottom), and reduced mobility (*μ*/*μ*_0_, bottom) for charged shell nanoparticles comprising a binary mixture of (1 – *z*) (PSMA_40_–PBzMA_2000_) and (*z*) ((P(SMA_36_-*stat*-MOTMA_4_))–PBzMA_2000_) copolymer chains in *n*-dodecane at a fixed volume fraction (1.5 × 10^–4^). The radii vary significantly depending on the cationic comonomer content, with a lower fraction of MOTMA-containing chains resulting in smaller nanoparticles. (The solid line is a linear fit as a guide to the eye.) The electrophoretic mobility is strongly dependent on the cationic comonomer content. For higher MOTMA contents, the electrophoretic mobility of the nanoparticles is equivalent to that of charged shell nanoparticles with maximum cationic content (*z* = 1). However, the electrophoretic mobility is equivalent to that of charged core nanoparticles at lower MOTMA contents. (The solid line is a linear fit to denote these regions.)

As an alternative to reducing the number of cationic units in the nanoparticle cores, the number of ionic groups in the shell was systematically reduced using a binary mixture of stabilizers comprising non-ionic and cationic PSMA-based macro-CTAs. The cationic stabilizer contains 10 mol% cationic comonomer (relative to PSMA), and this is reduced to approximately 0.50 mol% cationic comonomer when mixed with the highest proportion of non-ionic stabilizer used in these experiments. This results in nanoparticles that have the same effective number of cationic comonomer units in the stabilizer block as the smallest number of ionic groups incorporated into the particle cores (above and Fig. 10). Like the charged core nanoparticles, appreciable cationic character is observed in all cases for charged shell nanoparticles, even when containing relatively few cationic comonomers within the stabilizer chains (Fig. 11).

The charged shell nanoparticles remain electrophoretic at all MOTMA contents. However, unlike the charged core nanoparticles, the mobility depends on the cationic comonomer content. If the mol fraction of P(SMA_36_-*stat*-MOTMA_4_) (*z*) in a binary mixture of stabilizers is 0.6 or higher, the nanoparticles possess the same mobility as that expected for charged shell spheres at this volume fraction (Fig. 4). On the other hand, if *z* is below 0.3, the electrophoretic mobility of the nanoparticles is much greater and corresponds to that obtained for the equivalent charged shell nanoparticles. This suggests that the mobility of charged shell nanoparticles is reduced when cationic groups at the nanoparticle surface are located in close proximity. When the cationic comonomer content is lowered, repulsive interactions between cationic chains are reduced. The fact that the electrophoretic mobility actually increases as the number of ionic groups is reduced highlights the technical difficulty in accessing the low-charge limit in non-polar solvents.

The size of charged shell nanoparticles also strongly depends on their cationic comonomer content. This is not surprising given that such nanoparticles become significantly larger as a function of target PBzMA DP compared to uncharged spheres (Table 1). This is presumably the result of repulsion between charged stabilizer chains at the nanoparticle surface. Interestingly, for charged shell nanoparticles prepared using very low levels of cationic comonomer, the electrophoretic mobility is equivalent to that of charged core nanoparticles. The electrostatic interactions between stabilizer chains are clearly reduced for charged shell nanoparticles prepared with lower levels of cationic comonomer, as the particle size is equivalent to the non-ionic spheres and is significantly lower than for a charged core particle with the same electrophoretic mobility. Clearly, the spatial location of the cationic comonomer units is of primary importance in determining the electrokinetics.

## 4 Conclusions

A cationic comonomer can be incorporated into either the steric stabilizer block or the core-forming block of diblock copolymer nanoparticles during their RAFT-mediated PISA synthesis in *n*-dodecane. This enables the production of sterically-stabilized charged core or charged shell nanoparticles in salt-free media that differ only in the spatial location of the cationic comonomer. The precise control over the diblock copolymer architecture afforded by RAFT polymerization means that it is straightforward to insert ionic units selectively into either the nanoparticle core or stabilizer shell of these model particles. As far as we are aware, nanoparticles with ionic groups in the stabilizer block have not been previously synthesized in salt-free non-polar solvents. Such colloidal dispersions represent a new model system for studying electrokinetic behavior.

As demonstrated previously for water, alcohols, and non-polar solvents,^42^,^43^,^59^,^94^ RAFT-mediated PISA enables the systematic variation of various parameters. The ability to rationally control the size and morphology of nano-objects makes this approach ideal for developing new model electrophoretic colloids. For example, the particle size can be adjusted by varying the target DP of the core-forming block. However, the DP dependence on particle size differed according to the cationic comonomer content and spatial location. These charged spherical nanoparticles clearly undergo counterion condensation, resulting in the electrophoretic mobility depending strongly on both the particle radius and the particle volume fraction. Electrophoretic mobilities increased linearly with size for smaller particles but approached a plateau value for larger particles. Previous reports of the preparation of charged polymer particles in salt-free non-polar media employed conventional dispersion polymerization using free radical chemistry, which is best suited for producing relatively large particles.^39^ The ability of RAFT-mediated PISA to reproducibly and rationally target significantly smaller nanoparticles, as confirmed by microscopy and scattering studies, enables exploration of new regimes in which electrophoretic mobilities strongly depend on particle size. Interestingly, the magnitude of the mobility depends on the spatial location of the ionic groups: charged shell polymer nanoparticles had lower mobilities than charged core nanoparticles. These unexpected observations are anticipated to motivate further theoretical studies of charged shell nanoparticles in salt-free non-polar media. By varying the proportion of ionic comonomer, the difference in mobility between the charged core and charged shell nanoparticles appears to be the result of the relatively high concentration of ionic groups at the surface of the latter system. When charged shell nanoparticles were prepared with a relatively low fraction of stabilizer chains with ionic groups, the magnitude of the mobility was equivalent to charged core nanoparticles prepared with the same core block DP.

Finally, this study has revealed an unexpected aspect of the behavior of charged shell spheres in salt-free non-polar media, namely that their electrophoretic mobility is less than that observed for charged core spheres of equivalent size. New theoretical models of the electrokinetics of charged shell nanoparticles in salt-free non-polar media will be required to explain this observation, and these nanoparticles should serve as an ideal model system to test these theories.

## Conflicts of interest

There are no conflicts of interest to declare.

## Supplementary Material

Supplementary information

## References

[cit1] Pugh R. J., Matsunaga T., Fowkes F. M. (1983). Colloids Surf..

[cit2] Antl L., Goodwin J. W., Hill R. D., Ottewill R. H., Owens S. M., Papworth S., Waters J. A. (1986). Colloids Surf..

[cit3] Poon W. C. K. (2002). J. Phys.: Condens. Matter.

[cit4] Hsu M. F., Dufresne E. R., Weitz D. A. (2005). Langmuir.

[cit5] Leunissen M. E., Christova C. G., Hynninen A.-P., Royall C. P., Campbell A. I., Imhof A., Dijkstra M., van Roij R., van Blaaderen A. (2005). Nature.

[cit6] Bartlett P., Campbell A. I. (2005). Phys. Rev. Lett..

[cit7] Hudson L., Eastoe J., Dowding P. (2006). Adv. Colloid Interface Sci..

[cit8] Beunis F., Strubbe F., Neyts K., Petrov D. (2012). Phys. Rev. Lett..

[cit9] Gacek M. M., Berg J. C. (2015). Adv. Colloid Interface Sci..

[cit10] Binks B. P., Tyowua A. T. (2016). Soft Matter.

[cit11] Bjerrum N. (1926). Kgl. Dan. Vidensk. Selsk. Mat. Fys. Medd..

[cit12] Wohlfarth C., in CRC Handbook of Chemistry and Physics, CRC Press, 95th edn, 2014–2015, ch. Permittivity (Dielectric Constant) Of Liquids.

[cit13] Blagovidov I. F., Lapin V. P., Trofimpva G. L., Shor G. I. (1971). Chem. Technol. Fuels Oils.

[cit14] Electrostatics in the Petroleum Industry: The Prevention of Explosion Hazards, ed. A. Klinkenberg and J. L. van der Minne, Elsevier, London, 1958.

[cit15] Croucher M. D., Lok K. P., Wong R. W., Drappel S., Duff J. M., Pundsack A. L., Hair M. L. (1985). J. Appl. Polym. Sci..

[cit16] Comiskey B., Albert J. D., Yoshizawa H., Jacobson J. (1998). Nature.

[cit17] Heikenfeld J., Drzaic P., Yeo J.-S., Koch T. (2011). J. Soc. Inf. Disp..

[cit18] Novotny V. (1987). Colloids Surf..

[cit19] Morrison I. D. (1993). Colloids Surf., A.

[cit20] Smith G. N., Eastoe J. (2013). Phys. Chem. Chem. Phys..

[cit21] Smith G. N., Hallett J. E., Eastoe J. (2015). Soft Matter.

[cit22] Block H., Kelly J. P. (1988). J. Phys. D: Appl. Phys..

[cit23] Gast A. P., Zukoski C. F. (1989). Adv. Colloid Interface Sci..

[cit24] Hao T. (2001). Adv. Mater..

[cit25] Royall C. P., Leunissen M. E., van Blaaderen A. (2003). J. Phys.: Condens. Matter.

[cit26] Delgado A. V., Carrique F., Roa R., Ruiz-Reina E. (2016). Curr. Opin. Colloid Interface Sci..

[cit27] Manning G. S. (1969). J. Chem. Phys..

[cit28] Oosawa F., Polyelectrolytes, Marcel Dekker, New York, 1971.

[cit29] Manning G. S. (2002). Biophys. Chem..

[cit30] In the electrokinetics literature, charged core nanoparticles are referred to as “hard” charged spheres and charged shell nanoparticles as “soft” charged spheres

[cit31] Ohshima H. (2002). J. Colloid Interface Sci..

[cit32] Ohshima H. (2002). J. Colloid Interface Sci..

[cit33] Ohshima H. (2003). J. Colloid Interface Sci..

[cit34] Ohshima H. (2003). J. Colloid Interface Sci..

[cit35] Ohshima H. (2004). J. Colloid Interface Sci..

[cit36] Ohshima H. (2004). J. Colloid Interface Sci..

[cit37] Sánchez R., Bartlett P. (2011). Soft Matter.

[cit38] Hussain G., Robinson A., Bartlett P. (2013). Langmuir.

[cit39] Gillespie D. A. J., Hallett J. E., Elujoba O., Che Hamzah A. F., Richardson R. M., Bartlett P. (2014). Soft Matter.

[cit40] Royall C. P., Poon W. C. K., Weeks E. R. (2013). Soft Matter.

[cit41] Elsesser M. T., Hollingsworth A. D. (2010). Langmuir.

[cit42] Canning S. L., Smith G. N., Armes S. P. (2016). Macromolecules.

[cit43] Warren N. J., Armes S. P. (2014). J. Am. Chem. Soc..

[cit44] Fielding L. A., Derry M. J., Ladmiral V., Rosselgong J., Rodrigues A. M., Ratcliffe L. P. D., Sugihara S., Armes S. P. (2013). Chem. Sci..

[cit45] Fielding L. A., Lane J. A., Derry M. J., Mykhaylyk O. O., Armes S. P. (2014). J. Am. Chem. Soc..

[cit46] Derry M. J., Fielding L. A., Armes S. P. (2015). Polym. Chem..

[cit47] Lopez-Oliva A. P., Warren N. J., Rajkumar A., Mykhaylyk O. O., Derry M. J., Doncom K. E. B., Rymaruk M. J., Armes S. P. (2015). Macromolecules.

[cit48] Ratcliffe L. P. D., McKenzie B. E., Bouëdec G. M. D. L., Williams C. N., Brown S. L., Armes S. P. (2015). Macromolecules.

[cit49] Derry M. J., Fielding L. A., Warren N. J., Mable C. J., Smith A. J., Mykhaylyk O. O., Armes S. P. (2016). Chem. Sci..

[cit50] Cunningham V. J., Armes S. P., Musa O. M. (2016). Polym. Chem..

[cit51] Derry M. J., Mykhaylyk O. O., Armes S. P. (2017). Angew. Chem., Int. Ed..

[cit52] Houillot L., Bui C., Save M., Charleux B., Farcet C., Moire C., Raust J.-A., Rodriguez I. (2007). Macromolecules.

[cit53] Houillot L., Bui C., Farcet C., Moire C., Raust J.-A., Pasch H., Save M., Charleux B. (2010). ACS Appl. Mater. Interfaces.

[cit54] Raust J.-A., Houillot L., Save M., Charleux B., Moire C., Farcet C., Pasch H. (2010). Macromolecules.

[cit55] Pei Y., Thurairajah L., Sugita O. R., Lowe A. B. (2015). Macromolecules.

[cit56] Pei Y., Noy J.-M., Roth P. J., Lowe A. B. (2015). J. Polym. Sci., Part A: Polym. Chem..

[cit57] Pei Y., Sugita O. R., Thurairajah L., Lowe A. B. (2015). RSC Adv..

[cit58] Maiti B., Bauri K., Nandi M., De P. (2017). J. Polym. Sci., Part A: Polym. Chem..

[cit59] Derry M. J., Fielding L. A., Armes S. P. (2016). Prog. Polym. Sci..

[cit60] Canning S. L., Cunningham V. J., Ratcliffe L. P. D., Armes S. P. (2017). Polym. Chem..

[cit61] Obeng M., Milani A. H., Musa M. S., Cui Z., Fielding L. A., Farrand L., Goulding M., Saunders B. R. (2017). Soft Matter.

[cit62] Charbonnier A., Brochon C., Cloutet E., Navarro C., Hadziioannou G. (2013). J. Polym. Sci., Part A: Polym. Chem..

[cit63] Greinert N., Uerdingen M., Beylage L., Ignatyev N., Wilson J. H., Goulding M. J., Kemp R., Smith A. N., Bartlett P., Barthen P., Frank W. and Garcia R. S., Particles for electrophoretic displays, Patent WO 2012/072218 A1, 2012.

[cit64] Heenan R. K., Rogers S. E., Turner D., Terry A. E., Treadgold J., King S. M. (2011). Neutron News.

[cit65] Mantid Project, Mantid (2013): Manipulation and Analysis Toolkit for Instrument Data, 2013, 10.5286/software/mantid.

[cit66] Arnold O., Bilheux J. C., Borreguero J. M., Buts A., Campbell S. I., Chapon L., Doucet M., Draper N., Leal R. F., Gigg M. A., Lynch V. E., Markvardsen A., Mikkelson D. J., Mikkelson R. L., Miller R., Palmen K., Parker P., Passos G., Perring T. G., Peterson P. F., Ren S., Reuter M. A., Savici A. T., Taylor J. W., Taylor R. J., Tolchenov R., Zhou W., Zikovsky J. (2014). Nucl. Instrum. Methods Phys. Res., Sect. A.

[cit67] Wignall G. D., Bates F. S. (1987). J. Appl. Crystallogr..

[cit68] Doucet M., et al., SasView Version 4.1, Zenodo, 10.5281/zenodo.438138.

[cit69] Einstein A. (1906). Ann. Phys..

[cit70] Einstein A. (1911). Ann. Phys..

[cit71] Sigma-Aldrich, http://www.sigmaaldrich.com/united-kingdom.html.

[cit72] Schneider C. A., Rasband W. S., Eliceiri K. W. (2012). Nat. Methods.

[cit73] Ilavsky J., Jemian P. R. (2009). J. Appl. Crystallogr..

[cit74] Verduzco R., Li X., Pesek S. L., Stein G. E. (2015). Chem. Soc. Rev..

[cit75] Verduzco R., Li X., Pesek S. L., Stein G. E. (2015). Chem. Soc. Rev..

[cit76] Guinier A. (1939). Ann. Phys..

[cit77] Debye P. (1947). J. Phys. Chem..

[cit78] Pesek S. L., Li X., Hammouda B., Hong K., Verduzco R. (2013). Macromolecules.

[cit79] Hammouda B. (2010). J. Appl. Crystallogr..

[cit80] Hammouda B. (2010). J. Appl. Crystallogr..

[cit81] B. Hammouda, Probing Nanoscale Structures – The SANS Toolbox, http://www.ncnr.nist.gov/staff/hammouda/the\_SANS\_toolbox.pdf.

[cit82] Pedersen J. S., Schurtenberger P. (1996). Macromolecules.

[cit83] Chen W.-R., Butler P. D., Magid L. J. (2006). Langmuir.

[cit84] Tanford C., The Hydrophobic Effect: Formation of Micelles and Biological Membranes, John Wiley & Sons, Chichester, 2nd edn, 1980.

[cit85] Ohshima H. (2003). Colloids Surf., A.

[cit86] Pauw B. R. (2013). J. Phys.: Condens. Matter.

[cit87] Pedersen J. S., Gerstenberg M. C. (1996). Macromolecules.

[cit88] Pedersen J. S. (2000). J. Appl. Crystallogr..

[cit89] CRC, in CRC Handbook of Chemistry and Physics, CRC Press, 95th edn, 2014–2015, ch. Viscosity of liquids.

[cit90] The relative permittivities of the two solvents at 25 °C are essentially the same: *n*-dodecane is 2.006 and *n*-hexadecane is 2.040.12 On the other hand, the viscosities are very different at this temperature: *n*-dodecane is 1.383 mPa s and *n*-hexadecane is 3.032 mPa s.89

[cit91] Semsarilar M., Ladmiral V., Blanazs A., Armes S. P. (2012). Langmuir.

[cit92] Semsarilar M., Ladmiral V., Blanazs A., Armes S. P. (2013). Langmuir.

[cit93] Gonzato C., Semsarilar M., Jones E. R., Li F., Krooshof G. J. P., Wyman P., Mykhaylyk O. O., Tuinier R., Armes S. P. (2014). J. Am. Chem. Soc..

[cit94] Lowe A. B. (2016). Polymer.

