# Supplementary material for: Synthesis and electrokinetics of cationic spherical nanoparticles in salt-free non-polar media
Source: Chem Sci. 2017 Nov 17;9(4):922–34. doi: 10.1039/c7sc03334f (PMC5874696; doi:10.1039/c7sc03334f)
Supplement: Supplementary file 1 [file SC-009-C7SC03334F-s001.pdf]

Electronic Supplementary Material (ESI) for Chemical Science.
This journal is © The Royal Society of Chemistry 2017

Supporting Information—Cationic polymer spheres in salt-free nonpolar media

Gregory N. Smith,[*,†] Laura L. E. Mears,[‡] Sarah E. Rogers,[¶] and Steven P. Armes[§]

†*Department of Chemistry, University of Sheffield, Brook Hill, Sheffield, South Yorkshire, S3 7HF, United Kingdom*

‡*Department of Chemistry, University of Liverpool, Liverpool L69 7ZD, United Kingdom*

¶*ISIS-STFC, Rutherford Appleton Laboratory, Chilton, Oxon, OX11 0QX, United Kingdom*

§*Department of Chemistry, University of Sheffield, Brook Hill, Sheffield, South Yorkshire, S3 7HF, UK*

E-mail: g.n.smith@sheffield.ac.uk

Synthesis

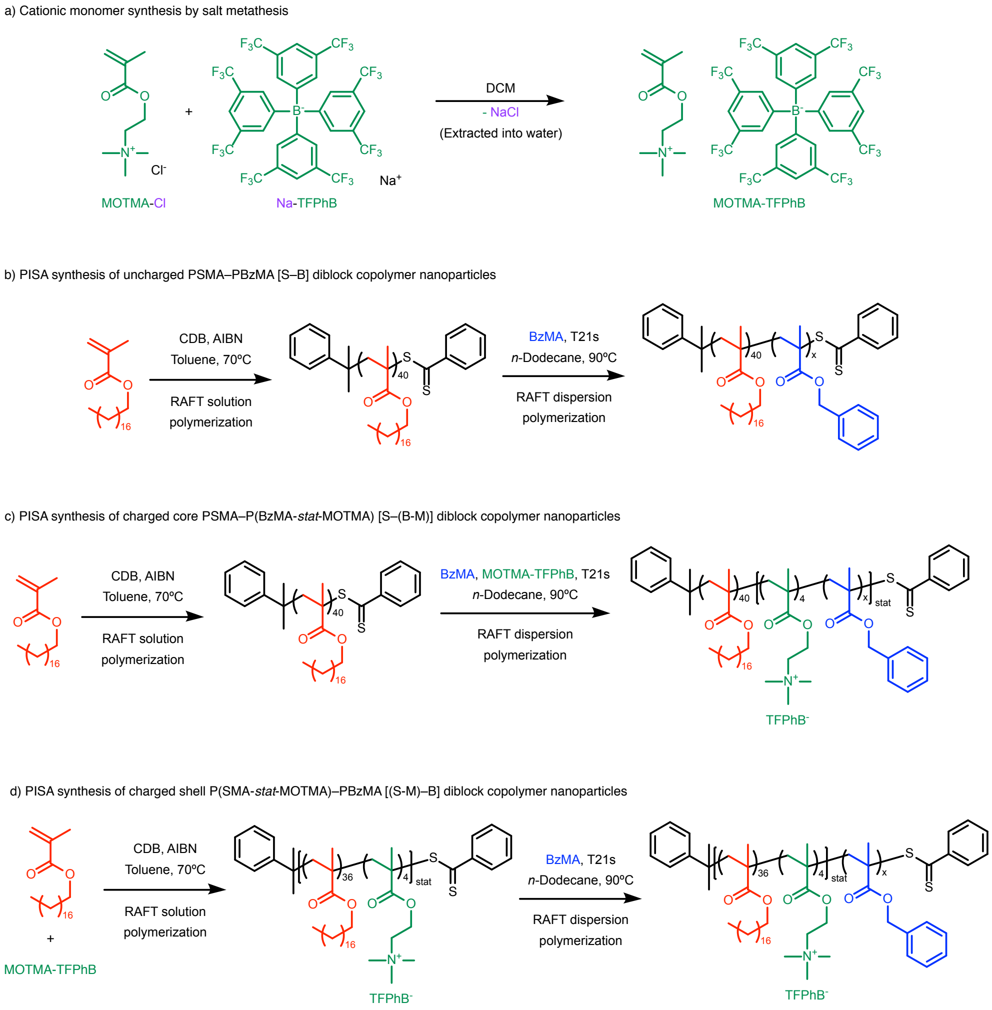

Scheme S1: (a) Metathesis reaction used to prepare the cationic monomer MOTMA-TFPhB. (b) PISA synthesis of uncharged diblock copolymers (solution polymerization of SMA followed by dispersion polymerization of BzMA). (c) PISA synthesis of charged core copolymers (solution polymerization of SMA followed by dispersion polymerization of BzMA and MOTMA-TFPhB). d) PISA synthesis of charged shell copolymers (solution polymerization of SMA and MOTMA-TFPhB followed by dispersion polymerization of BzMA). (Abbreviations for these copolymers used in figure legends are shown next to the copolymer names.)

Diblock copolymer characterization

Uncharged PSMA–PBzMA diblock copolymers

Table S1: Polymer characterization of $PSMA_{40}$–$PBzMA_y$

Target PBzMA DP (y)	Conversion	DP	M_n / (kg mol^{-1})	$Đ_M = M_w/M_n$
3651	75%	2758	89.1	5.96
2000	97%	1928	176.3	2.94
1095	95%	1084	124.7	3.00
600	98%	592	85.6	1.89
329	96%	322	58.6	1.62
180	96%	174	28.8	1.56

Table S2: Nanoparticle characterization of $PSMA_{40}$–$PBzMA_y$

Target PBzMA DP (y)	d_Z (n-Dodecane) / nm	d_Z (n-Hexadecane) / nm
3651	259	246
2000	159	153
1095	124	118
600	80.	79
329	71	70.
180	50.	48

Charged core PSMA–P(BzMA-*stat*-MOTMA) diblock copolymers

Table S3: Polymer characterization of PSMA–P(BzMA-*stat*-MOTMA) (PMOTMA DP ≈ 4)

Target PBzMA DP	BzMA Conversion	PBzMA DP	PMOTMA DP
3651	93%	3568	5
2000	89%	1808	5
1095	96%	1084	5
600	97%	586	4
329	96%	322	4
180	96%	169	4

Table S4: Nanoparticle characterization of PSMA–P(BzMA-*stat*-MOTMA) (PMOTMA DP ≈ 4)

Target PBzMA DP	d_Z (n-Dodecane) / nm	d_Z (n-Hexadecane) / nm
3651	382	374
2000	178	173
1095	122	118
600	84	85
329	65	64
180	46	46

Table S5: Characterization of PSMA–P(BzMA-*stat*-MOTMA) (PMOTMA DP < 4, Target PBzMA DP = 2000)

Target PMOTMA DP	BzMA Conversion	PBzMA DP	PMOTMA DP	d_Z / nm
2.8	95%	1857	3.7	174
1.4	96%	1957	1.1	168
0.7	80%	1626	0.6	170.
0.4	92%	1849	0.1	180.

Charged shell P(SMA-*stat*-MOTMA)–PBzMA diblock copolymers

Table S6: Polymer characterization of P(SMA-*stat*-MOTMA)–PBzMA

Target PBzMA DP	BzMA Conversion	PBzMA DP
3651	93%	3072
2000	97%	1898
1095	92%	1005
600	97%	594
329	95%	311
180	95%	164

Table S7: Nanoparticle characterization of P(SMA-*stat*-MOTMA)–PBzMA

Target PBzMA DP	d_Z (n-Dodecane) / nm	d_Z (n-Hexadecane) / nm
3651	285	276
2000	193	200
1095	133	128
600	93	91
329	77	75
180	66	51

Table S8: Characterization of [z P(SMA-*stat*-MOTMA) + $(1 - z)$ PSMA]–PBzMA (Target PBzMA DP = 2000)

macro-CTA ionic fraction	BzMA Conversion	PBzMA DP	d_Z / nm
0.85	93%	1939	175
0.70	94%	2008	217
0.57	94%	1790	168
0.51	86%	1679	154
0.44	90%	1812	153
0.32	86%	1758	158
0.15	92%	1832	152
0.10	95%	1837	155
0.05	90%	1890	150

SAXS model

The scattering cross section per unit volume from a dispersion of nanoparticles ($\mathrm{d}\Sigma/\mathrm{d}\Omega(Q)$), equivalently referred to as the intensity ($I(Q)$), can be given by the following expression, where $P(Q)$ is the form factor, $S(Q)$ is the structure factor, n is the number density, ϕ is the volume fraction, and V_t is the volume of the object.

$$\frac{\mathrm{d}\Sigma}{\mathrm{d}\Omega}(Q) \equiv I(Q) = nP(Q)S(Q) = \frac{\phi}{V_t}S(Q)P(Q) \tag{S1}$$

$P(Q)$ is related to the geometry of the scattering object. For composite objects, such as polymer micelles, $P(Q)$ depends on the scattering length density (SLD, ρ) difference between parts of the system as well as their volume, so is a function of both. $S(Q)$ accounts for deviations from random scattering, which only become appreciable for either concentrated dispersions or for strongly interacting species. The SAXS data reported here are for dilute dispersions, and in this case, $S(Q) = 1$.

The spherical nanoparticles studied here are diblock copolymer micelles, and the form factors of these have previously been reported in the literature.[1,2] The form factor (P_m) of a spherical diblock copolymer micelle consists of four terms: two self-terms (for the spherical core, P_s, and the chains on the surface, P_c) and two cross-terms (between the core and the chains, S_{sc}, and between different chains on the surface, S_{cc}).

$$P_m(Q) = N^2\beta_s^2 P_s(Q) + N\beta_c^2 P_c(Q) + 2N^2\beta_s\beta_c S_{sc}(Q) + N(N-1)\beta_c^2 S_{cc}(Q) \tag{S2}$$

In this expression, N is the aggregation number, and β_s and β_c are the total excess scattering lengths of blocks in the core and the shell, respectively. They are given by $\beta_s = V_s(\rho_s - \rho_m)$ and $\beta_c = V_c(\rho_c - \rho_m)$, where V is the volume of a block and ρ is the scattering length density of a block. ρ_m is the scattering length density of the solvent medium.

The form factor for the self-term is simply the well-known spherical form factor for a

sphere of radius r.[3,4]

$$P_s(Q) = \left[\frac{3\left[\sin(Qr) - Qr\cos(Qr)\right]}{(Qr)^3}\right]^2 \tag{S3}$$

The self-term for the chains in the corona given by the Debye function, assuming that they are Gaussian chains with a radius of gyration R_g.[5]

$$P_c(Q) = \frac{2\left[\exp(-Q^2R_g^2) - 1 + Q^2R_g^2\right]}{Q^4R_g^4} \tag{S4}$$

To mimic non-penetration of the Gaussian chains, they are set as starting a distance dR_g away from the surface of the core, where $d \approx 1$. The cross-term between core and chains is given by the following expression.

$$S_{sc}(Q) = \Phi(Qr)\psi(QR_g)\frac{\sin(Q\left[r + dR_g\right])}{Q\left[r + dR_g\right]} \tag{S5}$$

The functions $\Phi(x)$ and $\psi(x)$ are given below.

$$\Phi(x) = \frac{3\left[\sin(x) - Qr\cos(x)\right]}{(x)^3} \tag{S6}$$

$$\psi(x) = \frac{\left[1 - \exp(-x)\right]}{x} \tag{S7}$$

The interference term between chains in the corona is given by the following expression.

$$S_{cc}(Q) = \psi^2(QR_g)\left[\frac{\sin(Q\left[r + dR_g\right])}{Q\left[r + dR_g\right]}\right]^2 \tag{S8}$$

Two modifications have been to this standard model for spherical diblock copolymer micelles. First, A sigmoidal interface was assumed to account for a varying scattering length density at the micellar interface. The interface width σ was set to 2.5. This modified the interface by the term $\exp(-Q^2\sigma^2/2)$. Next, a radial profile was used to define scattering in the micelle corona using a linear combination of two cubic splines with fitting parameters cor-

responding to the width and weight coefficient. Further information on these modifications can be found elsewhere.[6–8]

The core radius of the micelles was fit with a Gaussian distribution with standard deviation σ_G, given in the expression below.

$$f_G(x) = \frac{1}{\sqrt{2\pi\sigma_G^2}} \exp\left(-\frac{(x-r)^2}{2\sigma_G^2}\right) \tag{S9}$$

SAXS data fitting

SAXS data were fit to models as explained in the previous sections. The core radius was allowed to vary, the stabilizer R_g was fixed, and the volumes and SLDs of the two blocks were fixed from the known mass densities of the materials.

The radii of gyration for the two PSMA stabilizers were calculated using an approach described by Derry *et al.*[8] The contour length of a PSMA or PMOTMA monomer is set to 2.55 Å (two C–C bonds in all-*trans* conformation), and the total contour length is the product of this length and the DP. The Kuhn length is considered to be equal to that of PMMA (15.3 Å). From these values, the unperturbed radius of gyration can be determined from $R_g = \sqrt{\mathrm{DP} \times 2.55 \times 15.3/6}$. This calculation gives an R_g for $PSMA_{40}$ of 16.1 Å and for $P(SMA_{36}$-*stat*-$MOTMA_4)$ of 15.3 Å. These values compare favorably to the radii of gyration determined from SANS measurements on these polymers as solutions in n-dodecane-d_{26}.

The volume of the polymer blocks are calculated from the mass density and the molar mass of the monomer unit, which is then multiplied by the DP of the unit to give a total block volume. The mass densities have been taking from the literature. For PSMA and PBzMA, these are available experimentally.[8,9] For $PMOTMA^+$, only the monomer density is available, and the polymer density was by scaling this by the ratio of the density of PMMA to MMA.[10] For $TFPhB^-$, the molecular volume was calculated by Hartree–Fock theory.[11] For n-dodecane, the mass density is available in the literature.[12] For the ionic species, the volumes are calculated assuming complete ion binding, as the dissociation constant for ions in non-polar solvents is extremely low.

Table S9: Mass densities of materials

	Mass density / (g cm^{-3})
PSMA	0.97
PBzMA	1.179
$PMOTMA^+$	1.40
$TFPhB^-$	1.95
n-Dodecane	0.7495

Using this mass density and the molar mass of the species, the molecular volume (V_m) of the polymer blocks can be calculated.

Table S10: Molecular volumes of polymer blocks

	Stabilizer block V_m / Å^3	Core block V_m / Å^3
$PSMA_{40}$–$PBzMA_{174}$	23176	43136
P(SMA_{36}-*stat*-$MOTMA_4$)–$PBzMA_{164}$	24580	40820
$PSMA_{40}$–P($BzMA_{169}$-*stat*-$MOTMA_4$)	23176	45815

To calculate the scattering length densities, both the coherent scattering length (b_i) and the molar volumes must be known.

$$\rho = \frac{\sum_i b_i}{V_m} \quad \text{(S10)}$$

For X-rays, as scattering arises from the interaction between X-rays and the atomic electron cloud, b_i is related to the atomic number. At X-ray energies away from absorption edges, the atomic scattering factor f_1 is well approximated by the atomic number. In this case, b_i is equal to the product of the atomic number and the classical electron radius (r_e).[13,14]

Table S11: X-ray scattering length densities of materials

	ρ_X / (10^{-6} Å^{-2})
PSMA	9.24
PBzMA	10.7
n-Dodecane	7.32

To fit the SAXS data, the molecular volume of the two blocks (Table S10), the scattering length densities (Table S11), and the PSMA R_g were fixed initially. The only modification to the these calculated values was the SLD of the stabilizer for the charged shell nanoparticles, which was allowed to vary and fit to a value slightly below the calculated value. This is likely due to the small fraction of anions that must be dissociated from the particles to make them electrophoretic. The best fit values are shown in Table S12 below along with the fit scattering curves in Figure S1.

Table S12: SAXS fitting parameters

	Scale	Background	r_c / Å (c_V)
$PSMA_{40}$–$PBzMA_{174}$	0.0125	0.014	135.7 (0.12)
P(SMA_{36}-*stat*-$MOTMA_4$)–$PBzMA_{164}$	0.0092	0.002	147.7 (0.13)
$PSMA_{40}$–P($BzMA_{169}$-*stat*-$MOTMA_4$)	0.0115	0.005	148.8 (0.11)

	ρ_c / (10^{-6} Å^{-2})	ρ_s / (10^{-6} Å^{-2})
$PSMA_{40}$–$PBzMA_{174}$	10.7	9.24
P(SMA_{36}-*stat*-$MOTMA_4$)–$PBzMA_{164}$	10.7	10.1
$PSMA_{40}$–P($BzMA_{169}$-*stat*-$MOTMA_4$)	11.1	9.24

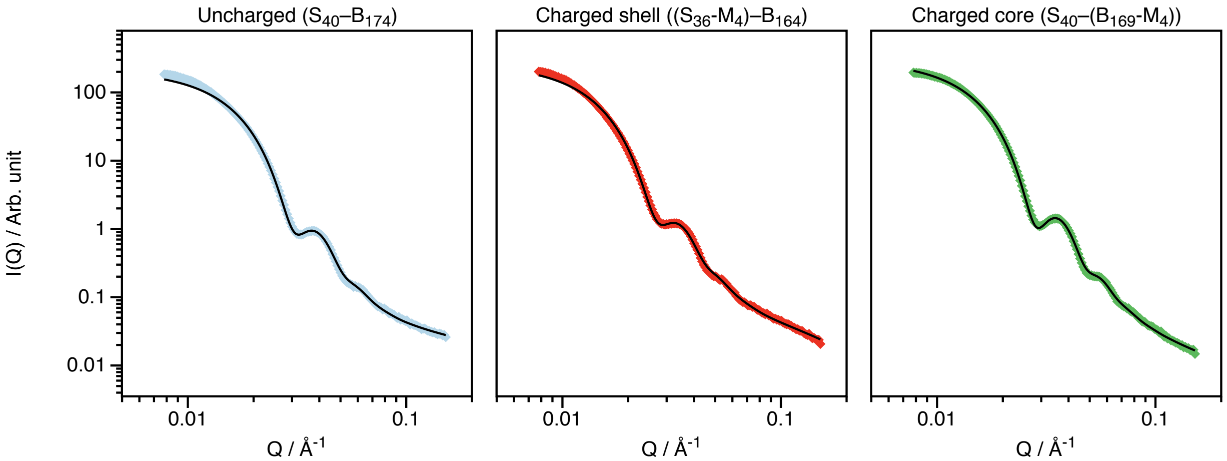

Figure S1: SAXS data for uncharged, charged shell, and charged core diblock copolymer nanoparticles. Experimental data has been fit to a diblock copolymer micelle model as explained in the text.

References

(1) Pedersen, J. S.; Gerstenberg, M. C. Scattering Form Factor of Block Copolymer Micelles. *Macromolecules* **1996**, *29*, 1363–1365.

(2) Pedersen, J. S. Form factors of block copolymer micelles with spherical, ellipsoidal and cylindrical cores. *J. Appl. Cryst.* **2000**, *33*, 637–640.

(3) Lord Rayleigh, The Incidence of Light upon a Transparent Sphere of Dimensions Comparable with the Wave-Length. *Proc. R. Soc. London A* **1910**, *84*, 25–46.

(4) Guinier, A.; Fournet, G. *Small-Angle Scattering of X-Rays*; John Wiley & Sons: New York, 1955.

(5) Debye, P. Molecular-weight Determination by Light Scattering. *J. Phys. Chem.* **1947**, *51*, 18–32.

(6) Pedersen, J. S.; Svaneborg, C.; Almdal, K.; Hamley, I. W.; Young, R. N. A Small-Angle Neutron and X-ray Contrast Variation Scattering Study of the Structure of Block Copolymer Micelles:âĂĽ Corona Shape and Excluded Volume Interactions. *Macromolecules* **2003**, *36*, 416–433.

(7) Pedersen, J. S.; Gerstenberg, M. C. The structure of P85 Pluronic block copolymer micelles determined by small-angle neutron scattering. *Colloids Surf. A: Physicochem. Eng. Aspects* **2003**, *213*, 175–187.

(8) Derry, M. J.; Fielding, L. A.; Warren, N. J.; Mable, C. J.; Smith, A. J.; Mykhaylyk, O. O.; Armes, S. P. *In situ* small-angle X-ray scattering studies of sterically-stabilized diblock copolymer nanoparticles formed during polymerization-induced self-assembly in non-polar media. *Chem. Sci.* **2016**, *7*, 5078–5090.

(9) Derry, M. J.; Mykhaylyk, O. O.; Armes, S. P. A Vesicle-to-Worm Transition Provides a

New High-Temperature Oil Thickening Mechanism. *Angew. Chem. Int. Ed.* **2017**, *56*, 1746–1750.

(10) Sigma–Aldrich. http://www.sigmaaldrich.com/united-kingdom.html.

(11) Frisch, M. J. et al. Gaussian 09 Revision D.01. Gaussian Inc. Wallingford CT 2009.

(12) CRC, *CRC Handbook of Chemistry and Physics*, 95th ed.; CRC Press, 2014–2015 (Internet Version).

(13) Scattering Length Density. http://gisaxs.com/index.php/Scattering_Length_Density.

(14) The Atomic Scattering Factor Files. http://henke.lbl.gov/optical_constants/asf.html.
